# Influence of Preterm Birth and Low Birthweight on Physical Fitness: A Systematic Review, Meta-Analysis, and Meta-Regression

**DOI:** 10.1007/s40279-024-02026-z

**Published:** 2024-05-06

**Authors:** Marcos D. Martínez-Zamora, Carlos Martín-Martínez, Óscar Martínez-de-Quel, Pedro L. Valenzuela

**Affiliations:** 1 Faculty of Education, Complutense University of Madrid, Madrid, Spain; 2 Faculty of Sciences for Physical Activity and Sport (INEF), Polytechnic University of Madrid, C/Martín Fierro, 7, 28040 Madrid, Spain; 3 Department of Systems Biology, University of Alcalá, Madrid, Spain; 4 Physical Activity and Health Research Group (PaHerg), Instituto de Investigación Hospital 12 de Octubre (‘imas12’), Centro de Actividades Ambulatorias, 7ª Planta, Bloque D, Av. de Córdoba s/n, 28041 Madrid, Spain

## Abstract

**Background:**

Preterm birth and low birthweight (LBW) might be associated with reduced physical fitness, although evidence remains inconclusive.

**Objective:**

To examine the influence of preterm birth and LBW on physical fitness, as well as to assess whether variables such as gestational age, birthweight, or age at assessment moderate these effects.

**Methods:**

PubMed, Scopus, and PsycINFO were systematically searched from inception to 7 December 2023 for case–control and cohort studies analyzing the association between preterm birth or LBW (or gestational age or birthweight as continuous variables) with at least one physical fitness-related outcome (i.e., cardiorespiratory fitness (CRF), muscle strength, flexibility, speed, agility). Random-effects meta-analysis and meta-regression models were used to estimate the pooled effect size, as well as to examine potential associations between the magnitude of the effect and gestational age, birthweight, or age at assessment.

**Results:**

Fifty-two studies (*n* = 920,603 participants, average age ranging from 4.7 to 34.4 years) were included. Preterm birth was associated with reduced CRF (standardized mean difference (SMD) = −0.38, 95% confidence interval (CI) = −0.51 to −0.25) and muscle strength (SMD = −0.44, 95% CI = −0.79 to −0.08). LBW was associated with reduced CRF (SMD = −0.40, 95% CI = −0.64 to −0.17), muscle strength (SMD = −0.18, 95% CI = −0.24 to −0.13), flexibility (SMD = −0.11, 95% CI = −0.22 to −0.01), and agility (SMD = −0.99, 95% CI = −1.91 to −0.07). Meta-regression analyses showed that a lower gestational age or birthweight were associated with larger reductions in physical fitness, whereas no consistent association was found for the age at assessment.

**Conclusion:**

Both preterm birth and LBW seem associated with reduced physical fitness regardless of age, with larger reductions overall observed in individuals with lower gestational age or birthweight. These findings might support the implementation of preventive strategies (e.g., fitness monitoring and physical exercise interventions) in these populations through the life course.

PROSPERO registration: CRD42021231845.

**Graphical Abstract:**

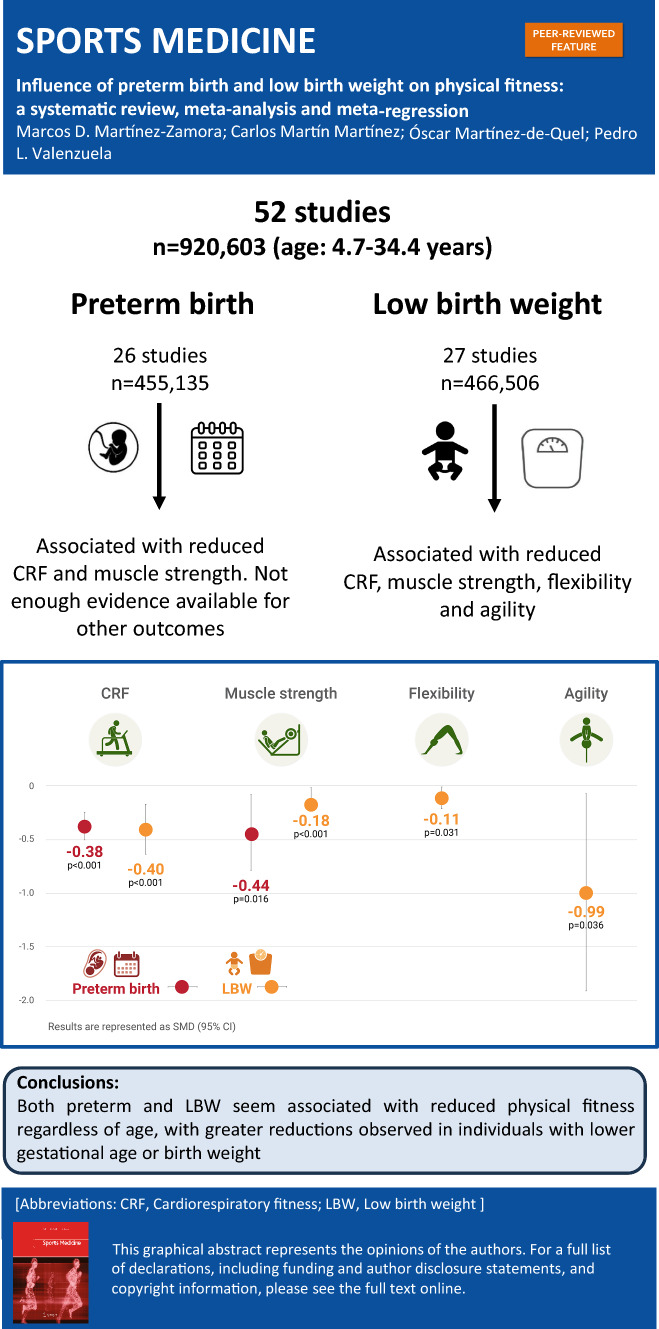

**Supplementary Information:**

The online version contains supplementary material available at 10.1007/s40279-024-02026-z.

## Key Points

**Table:** 

In this systematic review and meta-analysis, which included 52 studies (*n* = 920,603 participants, average age ranging from 4.7 to 34.4 years), both preterm birth and low birthweight were associated with reduced physical fitness (e.g., cardiorespiratory fitness, muscle strength).	
Meta-regression analyses revealed that a lower gestational age or birthweight were overall associated with larger reductions in physical fitness, whereas no consistent associations were found between participants’ age at assessment and the magnitude of the reductions in physical fitness (i.e., similar reduction from childhood to adulthood).	
These findings might support the implementation of preventive strategies (e.g., fitness monitoring, physical exercise interventions) in these populations through the life course.	

## Introduction

Preterm birth (conventionally defined as < 37 weeks of gestation) and low birthweight (LBW) (birthweight < 2.5 kg) are highly prevalent conditions [1, 2]. It is estimated that ~ 15 million children are born preterm every year, representing 10% of births worldwide [3], whereas approximately 20 million children are born with LBW, representing 15.5% of all births [4]—of note, although prematurity and LBW are two different conditions, they are highly interrelated, as many preterm individuals are born with LBW. Although advances in neonatal medicine have improved survival rates in preterm children and in those with LBW [5], these conditions are still linked to a higher risk of morbidity and mortality [6–8]. Indeed, one-quarter of all early deaths in newborns that are not produced by congenital malformations are due to preterm birth [9]. It is worth noting, however, that these conditions are not only associated with a higher morbidity risk during early childhood [10, 11], but also later in life. For instance, children with LBW have a 40-fold higher risk of mortality during the first month of life compared to those born with normal birthweight, with both preterm birth and LBW being linked to, for example, a higher risk of respiratory and cardiovascular conditions during adulthood [11–16].

Physical fitness (including different components such as cardiorespiratory fitness (CRF), muscle strength, speed, flexibility, or agility) is an important health indicator and a predictor of both short- and long-term morbidity and mortality risk in children and adolescents [17]. For instance, a reduced CRF during childhood is associated with a higher risk of developing conditions such as obesity and cardiometabolic diseases later in life [18, 19], and similar findings have been reported for muscle strength [20]. Moreover, CRF and muscle strength are strong predictors of morbidity and mortality in adults [21, 22].

A reduced physical fitness might play a role in the adverse effects associated with preterm birth and LBW. Growing evidence does indeed suggest that individuals born preterm [23, 24] or with LBW [25] present a reduced physical fitness, although meta-analytical evidence is scarce. In a meta-analysis of 22 studies, Edwards et al. [26] also found that preterm participants aged between 5 and 21 years had a lower maximum oxygen uptake (VO_2max_) compared with their peers born at term. Moreover, Dodds et al. [27] meta-analyzed 19 studies and found a positive association between birthweight and muscle strength, which was maintained across the life course. In a meta-analysis of ten studies, Poole et al. recently reported that participants aged over 18 years with LBW had a reduced CRF (as assessed by VO_2max_) compared with their term-born peers [28]. However, to our knowledge no meta-analytical evidence exists on the effect of preterm birth on muscle strength, nor for the effects of these conditions on other physical fitness outcomes such as flexibility, agility, or speed. Moreover, whether a lower birthweight or gestational age might be associated with greater reductions in physical fitness remains unclear, as well as whether the magnitude of these reductions might vary depending on the individuals’ age at assessment (e.g., with these differences decreasing at older ages).

The present systematic review and meta-analysis aimed to determine the effects of preterm birth and LBW on different physical fitness indicators, as well as to examine whether different variables (i.e., gestational age, birthweight, age at assessment) moderate these effects. Of note, although preterm birth and LBW are two interrelated conditions, we aimed to study them separately, which could help to understand the similarities and differences between them.

## Methods

The present systematic review was registered in PROSPERO (CRD42021231845), and is reported according to the Preferred Reporting Items for Systematic Reviews and Meta-analyses (PRISMA) [29].

### Study Selection and Search Strategy

Case–control and cohort studies analyzing the association between preterm birth or LBW (or gestational age or birthweight as a continuous variable) with at least one physical fitness-related outcome (i.e., CRF, muscle strength, flexibility, speed, or agility) were included. Studies were excluded if they were solely focused on specific populations such as individuals with overweight, respiratory conditions, or functional disabilities.

Two authors (MDMZ and CMM) independently performed the systematic search for relevant articles in PubMed, Scopus, and PsycINFO from inception up to 7 December 2023. The search included terms in titles and abstracts related to both the populations (i.e., preterm birth and LBW) and the outcomes of interest (i.e., physical fitness components). The search was limited to peer-reviewed articles published in English and Spanish. Additional search filters were not applied. The search strategies are shown as Table S1 in the Online Supplementary Material (OSM). The electronic search was supplemented with a manual review of reference lists from relevant publications and other reviews related to the topic [26–28] to locate additional studies.

Citations were first retrieved and preliminarily screened by title and abstract, and duplicates were removed manually. Full-texts of those studies that met the inclusion criteria were assessed. Each author provided a separate list with the studies selected at each stage, as well as with those to be finally included. Potential disagreements were resolved through discussion with two other authors (OMDQ and PLV).

### Data Extraction

Two authors (MDMZ and CMM) independently extracted the relevant information from each study (i.e., participants’ characteristics, outcomes assessed, and main results). Data for quantitative analyses were extracted, when available, as mean and standard deviation (SD). When data were provided as the median and/or using other measures of dispersion (e.g., standard error, range, 95% confidence interval (CI)), the required information was estimated as explained elsewhere [30]. When available, we used the most adjusted model (e.g., adjusting for covariates such as sex, age at assessment, or socioeconomic status) for analyses. We had to contact the authors of 14 studies because the required data were not reported. Of these, the authors of eight studies provided the required information [31–38].

### Study Quality Assessment

Study quality was determined using the Newcastle–Ottawa Scale (NOS) [39], which assesses the risk of bias considering three domains: selection of participants, comparability, and outcomes (Table S2, OSM). Two authors (MDMZ and CMM) independently scored the studies, and disagreements were resolved through discussion with a third author (OMDQ). A 0–10 total score was determined by counting the number of criteria satisfied by each study, which could be classified as having good (≥ 8), fair (7), or poor quality (≤ 6).

### Statistical Analysis

Pooled analyses were performed using a random-effects model (DerSimonian and Laird method) when at least three studies assessed a given outcome. The pooled standardized mean difference (SMD) between groups was computed along with 95% CI, and if the studies reported the same outcome using the same assessment method and measurement units (e.g., VO_2max_ in ml/kg/min, handgrip strength in kg), the absolute mean difference (MD) was computed. When a study assessed a given outcome at several time points, the longest follow-up was used for analyses. In the same line, when two studies shared some of the same participants, the study with the longest follow-up was analyzed. When two studies shared some of the same participants and had the same follow-up, the study with the largest sample size was used for analyses. For an initial general analysis, in those cases in which one study assessed different indicators related to the same outcome, we selected the outcome most commonly assessed (e.g., VO_2max_ over distance in the shuttle run test for the analysis of CRF, or handgrip strength over curl ups or horizontal jump distance for the analysis of muscle strength). However, sub-analyses were performed for the different indicators when possible. As recommended elsewhere, when a study included more than two groups of cases (e.g., extremely preterm and preterm individuals) in comparison with a control group, we halved the number of participants in the control group for each of the comparisons [30]. Sensitivity analyses were conducted by testing significance when removing one study at a time (leave-one-out method) to check if findings were mostly driven by an individual study. Meta-regression analyses were performed using a random effects model (method of moments) to assess the association between birthweight, gestational age, or age at assessment with the magnitude of the differences between groups. Meta-regression analyses were only performed for those outcomes assessed by ten or more studies [30]. Begg’s test was used to determine the presence of publication bias (small-study effects), and the *I*^2^ statistic was used to assess heterogeneity across studies. *I*^2^ values > 25%, 50%, and 75% were considered indicative of low, moderate, and high heterogeneity, respectively. The level of significance was set at 0.05. All statistical analyses were performed using the statistical software package Comprehensive Meta-analysis 2.0 (Biostat; Englewood, NJ, USA).

## Results

### Characteristics of the Included Studies

**Fig. 1:**
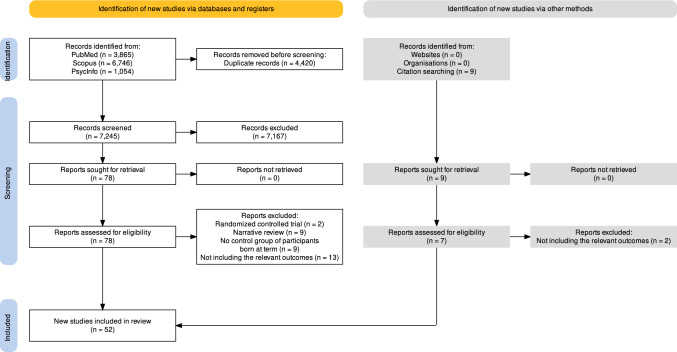
Flow diagram of the literature search

A flowchart of the literature search is shown in Fig. 1. A total of 52 studies (*n* = 920,603 participants) were eventually included, of which 26 (*n* = 455,135 participants) assessed the influence of preterm birth and 27 (*n* = 466,506 participants) were focused on the influence of LBW. In one study [40] all participants had both conditions. Some studies shared part of the same sample [41–43], and only the largest one was used for the computation of the total sample size to avoid duplication of participants. The characteristics of the included studies are summarized in Tables S3 and S4 (SM) for preterm and low birthweight participants, respectively.

The studies focused on preterm birth included between 21 and 218,802 participants with an average age ranging from 4.7 to 28 years (weighted average age 17.5 years), whereas those focused on LBW included between 30 and 144,369 participants with an average age ranging between 5 and 34.4 years (weighted average age 17.5 years). Most studies analyzed both male and female participants except for five studies that included solely male participants [23, 33, 34, 44, 45]. Most studies followed a cross-sectional case–control design, except for four cross-sectional cohort studies [33, 34, 46, 47]. Only three studies followed a longitudinal case–control design, in which the last measurement was considered for analyses [48–50]. Of the included case–control studies, eight divided the participants in sub-groups according to their gestational age or birthweight (e.g., very low birthweight and extremely low birthweight, extremely preterm and very preterm), whereas 40 of them combined them in one single group (i.e., controls vs. preterm or LBW). Most studies (*n* = 7) included participants from the USA, followed by Australia (*n* = 6), Norway (*n* = 4), and Sweden (*n* = 4). Most studies were conducted in medium- and high-income countries except for one [44] conducted in Mozambique.

### Quality Assessment

The quality of the included studies was overall good (average score of 8 out of 10, Table S5 (OSM)). Most studies adequately described the representativeness of the sample and justified the sample size (72% and 79%, respectively), although only 41% reported a satisfactory rate of response. All studies described the assessment tool and used a validated tool. Most studies (~ 93%) adjusted for the main demographic variables such as age and sex, and others also adjusted for socioeconomic status, physical activity levels, or body composition, but most of the studies did not adjust for potential confounding variables such as delivery mode or body mass index at the time of assessment. All studies described appropriately the statistical test performed, and 82% employed an independent blind assessment.

### Synthesis

A summary of the pooled results is shown in Table 1.

**Table 1:** Summary of pooled results

Outcome	Studies/groups (participants)	SMD (95% CI)	*p*-Value	*I*^2^	Begg’s *p*-value	Quality (mean NOS score, range)
Preterm birth						
CRF	17/24 (*n* = 89,230)	−0.38 (−0.51, −0.25)	** < 0.001**	77.4%	0.185	8 (6–10)
Muscle strength	8/10 (*n* = 2845)	−0.44 (−0.79, −0.08)	**0.016**	89.1%	0.036	8 (6–10)
Flexibility	2/2 (*n* = 216)	NA	NA	NA	NA	7 (7–7)
Agility	2/2 (*n* = 229)	NA	NA	NA	NA	6 (6–6)
Speed	1/1 (*n* = 60)	NA	NA	NA	NA	6 (6–6)
Low birthweight						
CRF	14/18 (*n* = 1758)	−0.40 (−0.64, −0.17)	**0.001**	76.1%	0.279	8 (6–10)
Muscle strength	10/16 (*n* = 274,100)	−0.18 (−0.24, −0.13)	** < 0.001**	86.6%	0.083	9 (7–10)
Flexibility	4/9 (*n* = 29,779)	−0.11 (−0.22, −0.01)	**0.031**	70.3%	0.038	9 (7–10)
Agility	3/7 (*n* = 29,695)	0.99 (−1.91, −0.07)	**0.036**	99.7%	0.500	9 (8–10)
Speed	2/2 (*n* = 506)	NA	NA	NA	NA	9 (8–10)

Results are shown as standardized mean difference (SMD) along with 95% confidence intervals (CIs)

Significant *p*-values are shown in bold font

*CRF* cardiorespiratory fitness, *NA* not applicable, *NOS* Newcastle Ottawa Scale

#### Cardiorespiratory Fitness (CRF)

Twenty-three studies [24, 37, 38, 40, 45, 49–66] assessed the influence of preterm birth on CRF-related measures such as VO_2max_, maximal power output (*W*_max_) on a cycle ergometer, distance covered on an incremental treadmill test, or the 6-min walking test. Of these, 17 studies including 24 group comparisons (*n* = 89,230 participants, weighted average age at assessment = 19 years) could be meta-analyzed. Pooled analyses revealed a reduced CRF in preterm individuals compared to controls (SMD = −0.38, 95% CI = −0.51 to −0.25, *p* < 0.001, Fig. S1), with no signs of risk of bias (Begg’s *p* = 0.185) but with large heterogeneity (*I*^2^ = 77.4%). Sensitivity analyses by removing one study at a time confirmed these differences, as well as when replacing the data from the 6-min walking test with data from the shuttle run test in the study by Cheong et al. [55], which analyzed both tests. Sub-analysis of those studies assessing VO_2max_ (11 studies, 15 group comparisons, *n* = 688 participants) also confirmed significantly lower values in individuals born preterm compared to controls (MD = −3.47 ml/kg/min, 95% CI = −5.04 to −1.89, *p* < 0.001; equivalent to SMD = −0.45, 95% CI = −0.61 to −0.28, *I*^2^ = 34.9%, Begg’s *p* = 0.444). Five studies could not be meta-analyzed [23, 46, 50, 58, 59], but only one of them did not find differences in CRF between preterm individuals and controls [59].

Seventeen [25, 34–36, 40–43, 47, 60, 67–73] studies assessed the influence of LBW on CRF-related measures, of which 14 including 18 group comparisons (*n* = 1758 participants, weighted average age at assessment = 18 years) could be meta-analyzed. Their pooled analysis revealed a reduced CRF in individuals born with LBW compared to controls (SMD = −0.40, 95% CI = −0.64 to −0.17, *p* = 0.001, Fig. S2 (OSM)), albeit with signs of heterogeneity (*I*^2^ = 76.1%). Sensitivity analyses confirmed these differences. All of them analyzed VO_2max_, and differences corresponded to a MD = −2.81 ml/kg/min (95% CI = −4.45 to −1.17, *p* = 0.001). Only two studies could not be meta-analyzed [34, 73], but both of them reported a significantly lower CRF among individuals with LBW.

#### Muscle Strength

Nine studies [24, 38, 46, 50, 58, 59, 74–76] assessed different muscle strength indicators such as standing long jump test, vertical jump, or handgrip strength with a dynamometer in preterm individuals. Of these, eight studies including ten group comparisons (*n* = 2845 participants, weighted average age at assessment = 17 years) could be meta-analyzed. The initial general analysis revealed a reduced muscle strength in preterm individuals compared to controls (SMD = −0.44, 95% CI = −0.79 to −0.08, *p* = 0.016, Fig. S3 (OSM)), with no signs of publication bias (Begg’s *p* = 0.059) but large heterogeneity (*I*^2^ = 89.1%). Sensitivity analyses revealed a significant or quasi-significant trend (all *p* ≤ 0.06) when removing each individual study. Sub-analysis of the five studies (*n* = 1843 participants) that assessed handgrip strength (in kg) confirmed significantly lower values in preterm individuals compared to controls in standardized (SMD = −0.19, 95% CI = −0.33 to −0.06, *p* = 0.004) and absolute units (MD = −0.64 kg, 95% CI = −1.01 to −0.27, *p* = 0.001), with no heterogeneity (*I*^2^ = 0%) and no signs of publication bias (Begg’s *p* = 0.226). One study could not be meta-analyzed [50], but found lower scores in extremely preterm participants compared to controls.

Ten studies [33, 35, 44, 47, 48, 69, 73, 77–79] including 16 group comparisons (*n* = 274,100 participants, weighted average age at assessment = 18 years) assessed the influence of LBW on muscle strength, and all of them could be meta-analyzed. A reduced muscle strength was overall observed in individuals with LBW compared to controls (SMD = −0.18, 95% CI = −0.24 to −0.13, *p* < 0.001, Fig. S4 (OSM)), albeit with signs of heterogeneity (*I*^2^ = 86.6%). Sensitivity analyses confirmed these differences. Sub-analysis of the seven studies (*n* = 145,299 participants) assessing handgrip strength also confirmed significantly lower values in individuals with LBW compared to controls (MD = −1.37 kg, 95% CI = −1.81 to −0.93, *p* < 0.001; equivalent to SMD = −0.23, 95% CI = −0.29 to −0.16), again with large heterogeneity (*I*^2^ = 86.4%) and no signs of publication bias (*p* = 0.296). Sub-analysis of the four studies (*n* = 129,112 participants) assessing lower-limb strength indicators such as vertical or horizontal jump tests also revealed significantly lower values in individuals with LBW compared to controls (SMD = −0.23, 95% CI = −0.38 to −0.08, *p* = 0.002), and the separate analysis of the three studies (*n* = 596 participants) that assessed vertical jump ability confirmed these differences (MD = −6.66 cm, 95% CI = −10.24 to −3.08, *p* < 0.001, equivalent to SMD = −0.74, 95% CI = −1.19 to −0.30), in this case with no heterogeneity (*I*^2^ = 0%) and no publication bias (*p* = 0.154).

#### Flexibility

Only data from two studies [24, 38] were available for the analysis of flexibility in preterm individuals, and therefore this outcome could not be meta-analyzed. However, only one of them found differences in flexibility related to preterm birth, with term individuals showing better scores [24].

Four studies [35, 44, 73, 78] including nine group comparisons (*n* = 29,779 participants, weighted average age at assessment = 7 years) assessed flexibility in individuals with LBW, all of them using the sit and reach test. Their pooled analysis revealed a reduced flexibility in individuals with LBW compared to controls (MD = −0.83 cm, 95% CI = −1.59 to −0.08, *p* = 0.031; equivalent to SMD = −0.11, 95% CI = −0.22 to −0.01, Fig. S5 (OSM)), albeit with large heterogeneity (*I*^2^ = 70.3%) and signs of publication bias (*p* = 0.038). Results remained significant in sensitivity analyses.

#### Agility

Only data from two studies [24, 58] were available for the meta-analysis of agility in preterm individuals, and therefore this outcome could not be analyzed. However, these studies reported that preterm birth is related to a reduced agility, as assessed by the 4 × 10 test.

On the other hand, three studies [35, 44, 78] including seven group comparisons (*n* = 29,695, weighted average age at assessment = 7 years) assessed agility in individuals with LBW using the 10 × 5 m shuttle test or the 4 × 4 m square test. Their pooled analysis revealed a reduced agility in individuals with LBW compared to controls (SMD = −0.99, 95% CI = −1.91 to −0.07, *p* = 0.036; Fig. S6 (OSM)), albeit with signs of large heterogeneity (*I*^2^ = 99.7%).

#### Speed

Only data from one [58] and two studies [35, 44] were available for the meta-analysis of speed (assessed by 20-m or 50-m running tests) in preterm and LBW individuals, respectively, and therefore this outcome could not be analyzed. These studies found a reduced speed among preterm individuals compared to controls, but no differences when analyzing LBW.

### Meta-Regressions

Meta-regression analyses could be performed on the influence of preterm birth and LBW on CRF and muscle strength. Analyses revealed a significant or quasi-significant trend towards a greater reduction of CRF in those individuals with a lower gestational age (*p* = 0.028, Fig. S7 (OSM)) or birthweight (*p* = 0.058, Fig. S8 (OSM)). Similarly, both a lower gestational age (*p* = 0.024, Fig. S9 (OSM)) and a lower birthweight (*p* < 0.001, Fig. S10 (OSM)) were associated with greater reductions in muscle strength. No significant associations were found in any case between the magnitude of the reductions in CRF or muscle strength and age at assessment (all *p* > 0.05, Figs. S11–S14 (OSM)).

## Discussion

**Fig. 2:**
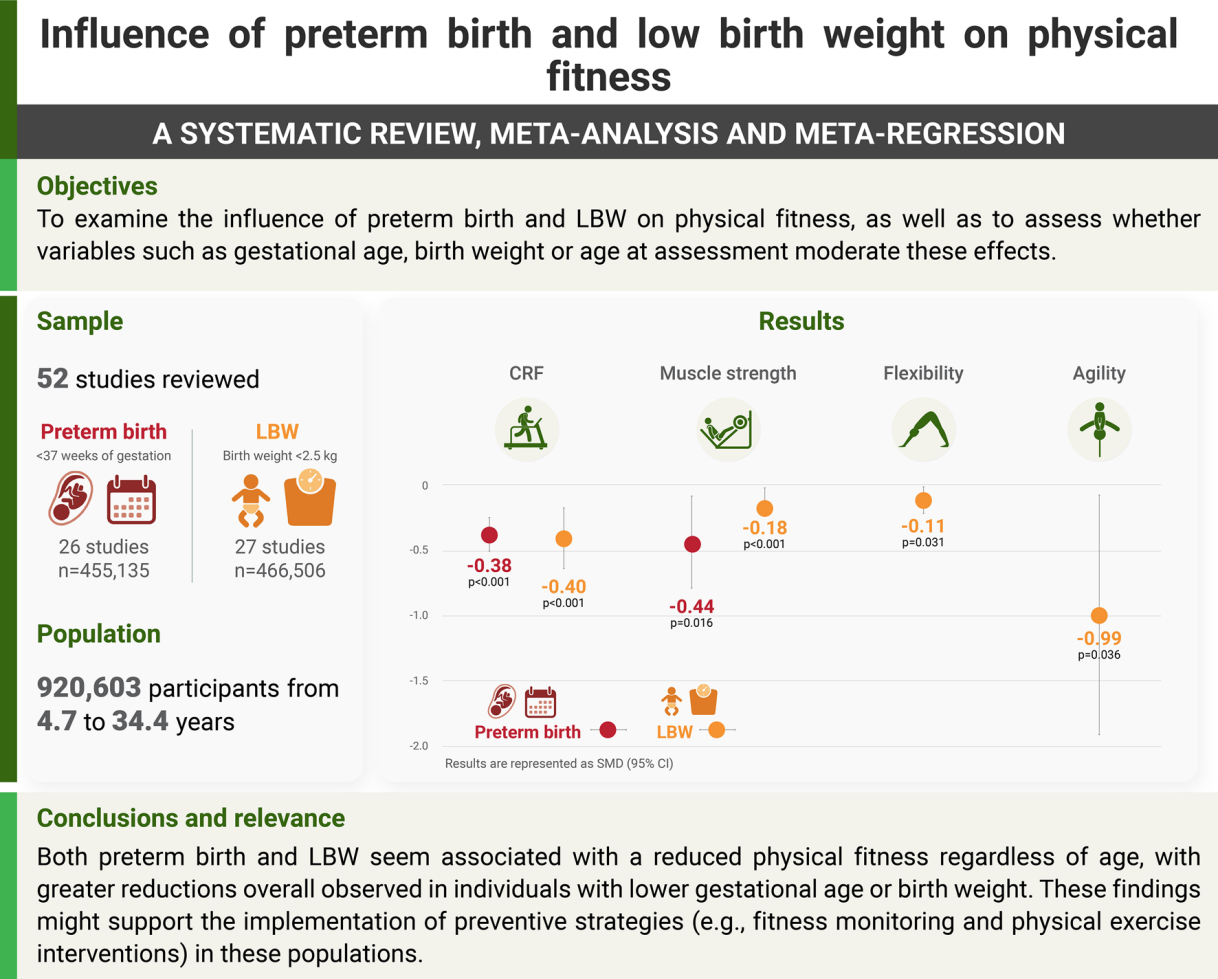
Graphical summary of the study findings

The present systematic review and meta-analysis [including 52 studies and 920,603 participants with a wide age range (from 4.7 to 34.4 years)] shows that both preterm birth and LBW are associated with a reduced physical fitness, as reflected by a lower CRF and muscle strength. Although the number of studies available precluded drawing strong conclusions on other fitness outcomes, our results also suggest that these conditions might also be associated with reductions in other fitness components, such as flexibility, or agility (which were reduced in individuals with LBW). Of note, a lower gestational age or birthweight seemed to be associated with greater reductions in physical fitness outcomes (at least for CRF and muscle strength, for which meta-regressions could be performed). No consistent associations were found between age at assessment and the magnitude of the reductions (see graphical summary of the results in Fig. 2).

The present findings are of potential clinical relevance due to the importance of physical fitness for both short- and long-term health. For instance, our results show that preterm individuals and those with LBW present a reduced CRF compared to controls (average differences in VO_2max_ of 4.29 and 3.10 ml/kg/min, respectively). A lower CRF during youth is associated with a higher risk of obesity and cardiometabolic diseases in later years [19], and indeed CRF has proven to be a major prognostic factor of mortality in individuals of all ages [22]. Interestingly, each metabolic equivalent reduction in CRF (i.e., 3.5 ml/kg/min, which is approximately the difference observed in the present study) has been associated with a 11.6%, 16.1%, and 14.0% increase in all-cause, cardiovascular, and cancer mortality, respectively [80], which further supports the potential relevance of our findings. Similar findings were observed for muscle strength levels in the present study, which were also reduced in individuals born preterm or with LBW. Low muscle strength levels during childhood are likely to be maintained into adulthood [81, 82], and are associated with a greater cardiometabolic risk later in life [20]. Indeed, muscle strength has been inversely associated with mortality risk in young [83] and adult populations [21]. Therefore, although further research is warranted to confirm the long-term clinical relevance of the observed differences, preventive strategies are needed to counteract the reduction of physical fitness observed in individuals born preterm or with LBW. In this regard, physical exercise might be an effective option, as shown by a recent study that reported increases in VO_2max_ in preterm individuals after 16 weeks of training [84].

The present findings expand on those from previous meta-analyses that provided preliminary evidence of reductions of physical fitness in individuals with LBW or preterm birth. Specifically, Edwards et al. found lower VO_2max_ values (−2.20 ml/kg/min) in preterm individuals compared to their counterparts aged between 5 and 21 years [26]. Poole et al. also reported that participants aged over 18 years with very low birthweight had lower VO_2max_ (−3.35 ml/kg/min) compared with their term-born peers [28]. In addition to confirming these findings, the present work suggests that preterm and LBW individuals might also show reductions in other major fitness outcomes such as muscle strength. This is in line with the meta-analysis by Dodds et al. [27], who reported a positive association between birthweight and muscle strength. Moreover, our results show reductions in other outcomes that to the best of our knowledge had not been previously meta-analyzed such as flexibility. Further research is, however, warranted to confirm whether other physical fitness components (e.g., agility, speed) are also reduced in these populations, as well as the mechanisms involved.

Another major finding of the present study derives from the meta-regression analyses. Similar to what has been reported for the risk of medical conditions [85], in the present study we found a trend toward greater reductions in physical fitness (specifically, CRF and muscle strength) with lower gestational age or birthweight. Confirming these trends, large cohort studies have also reported that birthweight is inversely associated with CRF and muscle strength [33, 34, 46]. Interestingly, our meta-regression analyses also suggest that the reductions in physical fitness associated with preterm birth or LBW seem not to be ameliorated at an older age, which is in line with the few longitudinal studies available on this topic. For instance, Pikel et al. [50] reported reductions in physical fitness components in preterm individuals after the period of childhood, some of which were maintained into early adulthood. Morrison et al. [48] found that individuals with LBW showed a similar change in grip strength from their mid-20s to their mid-30s, leading to consistently low levels in the former. Similarly, another longitudinal study reported that extremely preterm individuals showed a consistently lower physical fitness compared with those born at term through the school age, albeit only if they suffered from bronchopulmonary dysplasia [49]. Thus, preterm individuals not suffering from this condition progressively improved their fitness level through the school age, eventually reaching ‘normal’ values [49]. More research is therefore warranted to confirm these findings.

The present results might be at least partly explained by biological processes during pregnancy, which could be still evident at older ages. For instance, the association between gestational age and CRF might be explained by the late development of the lungs and cardiac chambers during pregnancy, which can lead to interrupted lung growth [86] in very preterm individuals. Dysanapsis might also be present in preterm individuals, that is, normal lung volumes and total cardiac size but smaller cardiac chambers and lung airways, which might impair expiratory airflow limitation [87]. Similarly, the reduction observed for other fitness outcomes such as muscle strength might also be related with essential processes in brain development that occur during the last weeks of gestation [88–90]. Moreover, although body composition was not analyzed in the present study, preterm individuals might also have a lower muscle mass and body mass index than their peers born at term [91], which might negatively influence fitness parameters such as muscle strength. It must be noted, nonetheless, that other behavioral or environmental factors such as the lower physical activity levels usually seen in preterm individuals might also be a confounding factor [92]. However, a recent study by our research group found reductions in several fitness components such as CRF, muscle strength, flexibility, and agility among preterm individuals compared to individuals born at term despite performing similar physical activity levels [24]. Thus, whether increasing physical activity levels can counteract the reduced fitness observed in individuals born preterm or with LBW remains to be elucidated.

### Limitations and Strengths

Some limitations of the present study should be acknowledged. The limited number of available studies for some outcomes such as speed or agility hindered performing meta-analyses. Moreover, meta-regression analyses could not be performed for all outcomes, as ten or more studies were required [30]. The required data from some studies could not be obtained despite asking the corresponding authors, which made it impossible to include these studies in quantitative synthesis. In addition, variables such as physical activity, body mass index, delivery mode, or socioeconomic status could potentially confound our findings, as most included studies did not adjust their analyses for these variables. Moreover, the inclusion of individuals with extremely low gestational age or birthweight could overestimate the observed results. Another limitation that should be considered is the fact that in the present review we did not assess the effects of being born small for gestational age, which should be addressed in future research.

On the other hand, the major strengths of this study are having analyzed two highly prevalent conditions such as preterm birth and LBW, which could help in understanding the similarities between them. In this regard, it is worth noting that these conditions are highly interrelated, and many individuals may present both of them concomitantly. Indeed, we observed rather similar estimates for outcomes such as CRF (SMD = −0.38 and −0.40 for preterm and LBW, respectively), although research is warranted to confirm whether there can be differences for other outcomes such as muscle strength. Moreover, the wide variety of fitness-related outcomes included and the inclusion of meta-regression analyses can also be considered strengths of our study. The large sample size, including participants with a wide age range from several cohorts of different countries, can also be considered a strength of the study, although the present findings might not be necessarily applicable to all populations, as only one study came from a low-income country.

## Conclusions

Both preterm birth and LBW seem to be associated with a reduced physical fitness, as reflected by lower values for outcomes such as CRF and muscle strength in an overall dose–response manner. Of note, the magnitude of these reductions seems overall independent of participants’ age at assessment, which suggests that these conditions are associated with reductions in physical fitness components not only during childhood, but also later in life. However, the magnitude of these reductions was inversely associated with both gestational age and birthweight. These findings might support the implementation of preventive strategies (e.g., fitness monitoring and lifelong exercise training) in these populations.

### Supplementary Information

Below is the link to the electronic supplementary material.

Supplementary file1 (DOCX 817 KB)

## Declarations

### Availability of Data and Material

Data are available upon request.
