# Supplementary material for: Influence of Preterm Birth and Low Birthweight on Physical Fitness: A Systematic Review, Meta-Analysis, and Meta-Regression
Source: Sports Med. 2024 May 6;54(7):1907–19. doi: 10.1007/s40279-024-02026-z (PMC11258071; doi:10.1007/s40279-024-02026-z)
Supplement: Supplementary file 1 — Supplementary file1 (DOCX 817 KB) [file 40279_2024_2026_MOESM1_ESM.docx]

**Table S1.** Search strategy (from inception up to December 7^th^, 2023).

PubMed/Medline

| Search | Query |
| --- | --- |
| #1 | (Fitness[Title/Abstract] OR balance[Title/Abstract] OR maximum oxygen uptake[Title/Abstract] OR maximum oxygen consumption[Title/Abstract] OR aerobic capacity[Title/Abstract] OR exercise capacity[Title/Abstract] OR strength[Title/Abstract] OR flexibility[Title/Abstract] OR agility[Title/Abstract] OR speed[Title/Abstract] OR cardiorespiratory capacity[Title/Abstract] OR endurance[Title/Abstract] OR motor skills[Title/Abstract] OR coordination[Title/Abstract]) |
| #2 | (premature birth[Title/Abstract] OR premature infants[Title/Abstract] OR premature children[Title/Abstract] OR premature bab*[Title/Abstract] OR preterm[Title/Abstract] OR low birth weight[Title/Abstract]) |
| #3 | #1 AND #2 |

PsycINFO and SCOPUS

| Search | Query |
| --- | --- |
| #1 | (Fitness[Title] OR balance[Title] OR maximum oxygen uptake[Title] OR maximum oxygen consumption[Title] OR aerobic capacity[Title] OR exercise capacity[Title] OR strength[Title] OR flexibility[Title] OR agility[Title] OR speed[Title] OR cardiorespiratory capacity[Title] OR endurance[Title] OR motor skills[Title] OR coordination[Title]) |
| #2 | (premature birth[Title] OR premature infants[Title] OR premature children[Title] OR premature bab*[Title] OR preterm[Title] OR low birth weight[Title]) |
| #3 | #1 AND #2 |

**Table S2.** Newcastle-Ottawa Scale (NOS).

|  | **Selection:** (Maximum 5 stars) |
| --- | --- |
| 1. | Representativeness of the sample (ordinary people):  a) Truly representative of the average in the target population. * (random sampling)  b) Somewhat representative of the average in the target population. * (non-random sampling)  c) Selected group of users.  d) No description of the sampling strategy. |
| 2 | Sample size:  a) Justified and satisfactory. *  b) Not justified. |
| 3 | Non-respondents:  a) Comparability between respondents and non-respondents characteristics is established, and the response rate is satisfactory. *  b) The response rate is unsatisfactory, or the comparability between respondents and non-respondents is unsatisfactory.  c) No description of the response rate or the characteristics of the responders and the non-responders. |
| 4 | Ascertainment of the exposure (prematurity or low birth weight):  a) Verification of the clinical record. **  b) Family information about the exposure. *  c) No description of the measurement tool. |
|  | **Comparability:** (Maximum 2 stars) |
| 5 | The subjects in different outcome groups are comparable, based on the study design or analysis. Confounding factors are controlled (sex and age).  a) The study compares participants with a similar age and sex, or controls for these two factors (sex and age). **  b) The study controls only for sex or age or for any additional factor. *  c) No controlling for any factors or no description. |
|  | **Outcome:** (Maximum 3 stars) |
|  | 1) Assessment of the outcome (fitness):  a) Independent blind assessment. **  b) Record linkage. *  c) No description.  2) Statistical test:  a) The statistical test used to analyze the data is clearly described and appropriate to compare PT/LBW participants with controls, and the measurement of the association is presented, including confidence intervals and the probability level (p value). *  b) The statistical test is not appropriate, not described or incomplete. |

**Table S3:** Summary of the studies included in the systematic review analyzing the influence of preterm birth (or gestational age as continuous variable) on physical fitness.

| *Authors* | *Design* | *Population* | | *Outcomes (test)* | *Main results* | |
| --- | --- | --- | --- | --- | --- | --- |
| Bruun et al.^1^ | Case-control | CASES:  Sample (N, sex): 38, (42% male).  Age (mean ± SD): 23.6 years ± 0.7.  GA (mean ± SD): 30.5 ± 1.3 weeks.  BW (mean ± SD): 1397 ± 300 g. | CONTROLS:  Sample (N, sex): 39, (41% male).  Age (mean ± SD): 24.3 ± 0.8 years.  GA (mean ± SD): 39.2 ± 1.1 weeks.  BW (mean ± SD): 3517 ± 470 g. | Strength:  Dynamometer | ↓Grip strength in preterm females. | |
| Caskey et al.^1^ | Case-control | CASES:  Sample (N, sex): 49, (57% male).  -Non-BPD group: 24, (58% male).  -BPD group: 25, (56% male).  Age (mean ± SD):  -Non-BPD group: 26.4 ± 3.7 years.  -BPD group: 24.0 ± 3.4 years.  GA (mean ± SD):  -Non-BPD group: 30.6 ± 1.9 weeks.  -BPD group: 26.8 ± 2.3.  BW (mean ± SD):  -Non-BPD group: 1234 ± 205 g.  -BPD group: 866 ± 255 g. | CONTROLS:  Sample (N, sex): 25, (60% male).  Age (mean ± SD): 28.3 ± 3.3 years.  GA (mean ± SD): 38.5 ± 0.9 weeks.  BW (mean ± SD): 3569 ± 297 g. | CRF:  Treadmill test. | ↓ VO_2max_. | |
| Cheong et al.^2^ | Case-control | CASES:  Sample (N, sex): 165, (45% male).  Age (mean): 25 years.  GA (mean ± SD): 26.6 ± 2 weeks.  BW (mean ± SD): 880 ± 155 g. | CONTROLS:  Sample (N, sex): 127, (43% male).  Age (mean): 25 years.  GA (mean ± SD): 39.3 ± 1.3 weeks.  BW (mean ± SD): 3387 ± 461 g. | CRF:  Shuttle run test. | ↓VO_2max_. | |
| Cousins et al.^3^ | Case-control | CASES:  Sample (N, sex): 116, (51% male).  Age (mean, range): 11.1, (10.8-11.3) years.  GA (mean, range): 30.9, (30.4-31.4) weeks.  BW (mean, range): 1690, (1585-1795) g. | CONTROLS:  Sample (N, sex): 70, (53% male).  Age (mean, range): 10.5, (10.2-10.7) years.  GA (mean, range): 40.0, (39,7-40.3) weeks.  BW (mean, range): 3528, (3403-3651) g. | CRF:  Cycle ergometer. | ↓CRF. | |
| Fitzgerald et al.^4^ | Case-control | CASES:  Sample (N, sex): 123, (49% male).  Age (mean): 4.7 years.  GA (mean ± SD): 27.8 ± 1.5 weeks.  BW (mean ± SD): 1037 ± 260 g. | CONTROLS:  Sample (N, sex): 128, (48% male).  Age (mean): 4.9 years.  GA (mean ± SD): 39.9 ± 1.2 weeks.  BW (mean ± SD): 3490 ± 445 g. | Strength:  Dynamometer. | ↓Grip strength. | |
| Goss et al.^5^ | Case-control | CASES:  Sample (N, sex): 11, (45% male).  Age (mean ± SD): 27.3 ± 0.8 years.  GA (mean ± SD): 28.6 ± 2.7 weeks.  BW (mean ± SD): 1087 ± 297 g. | CONTROLS:  Sample (N, sex): 10, (50% male).  Age (mean ± SD): 26.3 ± 0.9 years.  GA (mean ± SD): at term.  BW (mean ± SD): NR. | CRF:  Cycle ergometer. | ↓ VO_2max_. | |
| Huckstep et al.^6^ | Case-control | CASES:  Sample (N, sex): 47, (30% male).  Age (mean ± SD): 22.7 ± 3 years.  GA (mean ± SD): 32.8 ± 3.2 weeks.  BW (mean ± SD): 1916 ± 806 g. | CONTROLS:  Sample (N, sex): 54, (48% male).  Age (mean ± SD): 23.6 ± 3.8 years.  GA (mean ± SD): 39.5 ± 1.4 weeks.  BW (mean ± SD): 3390 ± 424 g. | CRF:  Cycle ergometer. | ↓ VO_2max_. | |
| Kosiecz et al.^7^ | Case-control | CASES:  Sample (N, sex): 30, (26% male).  Age (mean): 7 years.  GA (mean): 29.6 weeks.  BW (mean): NR. | CONTROLS:  Sample (N, sex): 30, (53% male).  Age (mean): 7 years.  GA (mean ± SD): NR.  BW (mean ± SD): NR. | CRF:  600-meter run.  Strength:  Long jump, grip strength, abdominal muscle strength and bar hang.  Agility:  4x10 test.  Speed:  50 m test. | ↓Grip strength, agility, speed and CRF. | |
| Kriemler et al.^9^ | Case-control | CASES:  Sample (N, sex): 14 (57% male).  Age (range): 6.1-7.8 years.  GA (mean ± SD):  28.3 ± 1.7 weeks.  BW (mean ± SD):  -1,108 ± 202 g. | CONTROLS:  Sample (N, sex): 24 (65% male).  Age (range): 5.5-7.4 years.  GA (mean ± SD): NR.  BW (mean ± SD): 1455 ± 595 g. | CRF:  Cycle ergometer. | ↓CRF. | |
| Lovering et al.^8^ | Case-control | CASES:  Sample (N): 24.  -Non-BPD group: 12.  -BPD group: 12.  Age (mean ± SD):  -Non-BPD group: 21.1 ± 3.5 years.  -BPD group: 23.8 ± 4.0 years.  GA (mean ± SD):  -Non-BPD group: 27.5 ± 1.9 weeks.  -BPD group: 28.6 ± 2.3.  BW (mean ± SD):  -Non-BPD group: 1160 ± 450 g.  -BPD group: 1040 ± 250 g. | CONTROLS:  Sample (N): 12.  Age (mean ± SD): 23.2 ± 4.0 years.  GA (mean ± SD): NR.  BW (mean ± SD): NR. | CRF:  Cycle ergometer. | No differences in the relevant outcomes. | |
| Martínez- Zamora et al.^9^ | Case-control | CASES:  Sample (N, sex): 98, (55% male).  Age (mean ± SD): 5.1 ± 0.8 years.  GA (mean ± SD): 32.4 ± 2.3 weeks.  BW (mean ± SD): 1725 ±508 g. | CONTROLS:  Sample (N, sex): 74, (53% male).  Age (mean ± SD): 4.8 ± 0.9 years.  GA (mean ± SD): 39.9 ± 1.0 weeks.  BW (mean ± SD): 3336 ± 392 g. | CRF:  Shuttle run test.  Strength:  Horizontal jump and dynamometer.  Agility:  4x10 test.  Flexibility:  Adapted sit and reach. | ↓CRF, muscle strength (upper and lower limbs), agility and flexibility. | |
| Mckay et al.^10^ | Case-control | CASES:  Sample (N, sex): 32, (37% male).  Age (mean, SD): 18.2 ± 6.9 years.  GA (range): <32 weeks.  BW (range): <1500 g. | CONTROLS:  Sample (N, sex): 40, (45% male).  Age (mean ± SD): 19.7 ± 6.5 years.  GA (range): >32 weeks.  BW (range): >1500g. | CRF:  Cycle ergometer. | ↓VO_2max_. | |
| Narang et al.^11^ | Case-control | CASES:  Sample (N, sex): 14, (NR male).  Age (mean ± SD): 9.5 ± 0.3 years.  GA (mean ± SD): 29.3 ± 1.8 weeks.  BW (mean ± SD): 1202 ± 184 g. | CONTROLS:  Sample (N, sex): 15, (NR male).  Age (mean ± SD): 9.7± 0.9 years.  GA (mean ± SD): 39.3 ± 1.3 weeks.  BW (mean ± SD): 3270 ± 307 g. | CRF:  Cycle ergometer. | No differences in the relevant outcomes. | |
| O’Dea et al.^12^ | Case-control | CASES:  Sample: 73  -BPD group (N, sex): 38, (47% male).  -No BPD group (N, sex): 35, (66% male).  Age (mean ± SD): 11 ± 0.6 years.  GA (mean ± SD):  -BPD group: 26.3 ± 2.4 weeks.  -No BPD group: 30 ± 1.55 weeks.  BW (mean ± SD):  -BPD group: 821 ± 207 g.  -No BPD group: 1435 ± 228 g. | CONTROLS:  Sample (N, sex): 31, (32% male).  Age (mean ± SD): 10.9 ± 0.7 years.  GA: >32 weeks.  BW: NR. | CRF:  Cycle ergometer. | No differences in the relevant outcomes. | |
| Pikel et al.^13^ | Case-control | CASES:  Sample (N, sex): 178, (55% male).  Age (mean): tested each year from 8-18.  GA (mean ± SD):  -VPT group girls: 28.7 ± 2.3 weeks.  -VPT group boys: 29.5 ± 1.8 weeks.  -MPT group girls: 34.9 ± 1.3 weeks.  -MPT group boys: 34.9 ± 1.2 weeks.  BW (mean ± SD):  -VLBW group girls: 1050 ± 240 g.  -VLBW group boys: 1230 ± 200 g.  -MLBW group boys: 2080 ± 290 g.  -MLBW group girls: 2100 ± 250 g. | CONTROLS:  Sample (N, sex): 218, (57% male).  Age (mean): tested each year from 8-18.  GA (mean ± SD):  -Girls: 39.5 ± 1.1 weeks.  -Boys: 39.5 ± 1.2 weeks.  BW (mean ± SD):  -Girls: 3300 ± 450 g.  -Boys: 3370 ± 470 g. | CRF:  600 m test.  Strength:  Horizontal jump and bend arm test.  Speed:  Plate Tapping. | ↓ Speed and VO_2max_.  No differences in strength. | |
| Pinheiro et al.^14^ | Case-control | CASES:  Sample (N, sex): 34, (50% male).  Age (mean ±SD): 8 ± 1.0 years.  GA (mean ± SD): 29.8 ± 2.5 weeks.  BW (mean ± SD): 1131± 228 g. | CONTROLS:  Sample (N, sex): 31, (58% male).  Age (mean ±SD): 7.9 ± 1.1 years.  GA (mean ± SD): 38.5 ± 1.4 weeks.  BW (mean ± SD): 3072 ± 609 g. | CRF:  Cardiopulmonary exercise test (CPET) and symptom-limited treadmill test. | No differences in the relevant outcomes. | |
| Praprotnik et al.^15^ | Case-control  (longitudinal) | CASES:  Sample (N, sex): 50,  Age (mean ± SD): 7.6 ± 0.9 years.  GA (mean ± SD): 26.8 ± 2 weeks.  BW (mean ± SD): 960 ± 260 g. | CONTROLS:  Sample (N, sex): 30,  Age (mean ± SD): 8.0 ± 1.0 years.  GA (mean ± SD): more than 36 weeks.  BW (mean ± SD): NR. | Physical fitness  A compound of eight different tests. | No differences in the relevant outcomes, except for preterm children with bronchopulmonary dysplasia. | |
| Ruf et al.^16^ | Case-control | CASES:  Sample (N, sex):22, (52% male).  -BPD group: 9, (44% male).  -Non-BPD group: 13, (54% male).  Age (mean ± SD):  -BPD group: 10.9 ± 1.7 years.  -Non-BPD group: 10.4 ± 1.5 years.  GA (mean ± SD; range):  -BPD group: 26.6 ± 1.6; 24.6–29.7 weeks.  -Non-BPD group: 29.1 ± 1.8; 25.0–31.0 weeks.  BW (mean ± SD; range):  -BPD group: 766.7 ± 212.4; 450–1080 g.  -Non-BPD group: 1117.3 ± 242.6; 780–1440 g. | CONTROLS:  Sample (N, sex):15, (53% male).  Age (mean ± SD): 9.9 ± 1.3 years.  GA (mean ± SD; range):39.3 ± 0.9 weeks; 38.0–41.0.  BW (mean ± SD; range): 3383 ± 328; 2800–3950g. | CRF:  Cycle ergometer. | No differences in the relevant outcomes. | |
| Svedenkrans et al.^17^ | Case-control | CASES:  Sample (N, sex): 10.709, (100% male).  Age (mean; range): 18 years; 18-26.  GA (range):  -56 with ≤ 28 GA.  -726 between 28 and 31 GA.  -9927 between 32 and 36 GA.  BW (mean ± SD): NR. | CONTROLS:  Sample (N, sex): 208093, (100% male).  Age (mean; range): 18 years; 18-26.  GA (mean ± SD):  -182477 between 37 and 42 GA.  -25616 with more than 42 GA.  BW (mean ± SD): NR. | CRF:  Cycle ergometer. | ↓ VO_2max._ | |
| Svedenkrans et al.^18^ | Case-control | CASES:  Sample (N, sex): 10712, (100% male).  Age (mean; range): 18 years; 18-26.  GA (range):  -56 with ≤ 27 GA.  -726 between 28 and 31 GA.  -9930 between 32 and 36 GA.  BW (mean ± SD): NR. | CONTROLS:  Sample (N, sex): 208108, (100% male).  Age (mean, range): 18 years; 18-26.  GA (mean ± SD):  -182490 between 37 and 42 GA.  -25618 ≥ 42 GA  BW (mean ± SD): NR. | CRF:  Cycle ergometer. | ↓ VO_2max._ | |
| Svien^19^ | Case-control | CASES:  Sample (N, sex): 22, (50% male).  Age (mean; range): 8.5; 7-10.8 years.  GA (mean; range): 32; 30-35 weeks.  BW (mean; range): 1884; 1248-2460 g. | CONTROLS:  Sample (N, sex): 22, (50% male).  Age (mean; range): 8.6; 7-11.3 years.  GA (mean; range): 40; 38-40 weeks.  BW (mean; range): 3435; 2700-4460 g. | CRF:  Treadmill test.  Strength:  Bruininks-Oseretsky Test of Motor Proficiency (BOTMP)  Flexibility:  Sit and reach, elbow and knee extension, from thumb to wrist. | ↓ Strength.  No significant differences for the remaining outcomes of interest. | |
| Tikanmaki et al.^20^ | Case-control | CASES:  Sample (N, sex): 386, (48% male).  -EP group: 139, 74F/65M.  -LP group: 247, 127F/120M.  Age (mean ± SD):  -EP group: 23.1 ± 1.3 years.  -LP group: 23.1 ± 1.4 years.  GA (mean ± SD):  -EP group: 31.8 ± 2 weeks.  -LP group: 35.8 ± 0.8 weeks.  BW (mean ± SD):  -EP group: 1784 ± 486 g.  -LP group: 2668 ± 514 g. | CONTROLS:  Sample (N, sex): 352, (48% male).  Age (mean ± SD): 23.6 ± 1.1 years.  GA (mean ± SD): 40.1 ± 1.2 weeks.  BW (mean ± SD): NR. | CRF:  Heart rate after a 4 minutes steps test.  Strength:  Modified push-ups test in 40 seconds and dynamometer strength. | ↓ Strength.  No significant differences for the remaining outcomes of interest. | |
| Van Deutekom et al.^21^ | Cohort | Sample (N, sex): 194, (54% male).  Age (mean ± SD): 8.6 ± 0.4 years.  GA (mean ± SD): 39.7 ± 2.1 weeks.  BW (mean ± SD): NR. | | CRF:  Shuttle run test.  Strength:  Grip strength with a dynamometer and horizontal jump. | Lower birth weight (as a continuous variable) associated with impaired VO_2max_ and strength. | |
| Vrijlandt et al.^22^ | Case-control | CASES:  Sample (N, sex): 42, (50% male)  Age (mean ± SD; range): 19 ± 0.3; 19-20 years.  GA (mean ± SD; range): 30 ± 2; 26-36 weeks.  BW (mean ± SD; range): 1246 ± 232; 720-1750 g. | CONTROLS:  Sample (N, sex): 48, (33% male).  Age (mean ± SD; range): 20.8 ± 1.2; 18-22 years.  GA (mean ± SD; range): at term; 37-42 weeks.  BW (mean ± SD): Not Applicable. | CRF:  Cycle ergometer. | No differences in the relevant outcomes. | |
| Weigelt et al.^23^ | Case-control | CASES:  Sample (N, sex): 33, (51% male).  Age (mean ± SD): 9.2 ± 0.6 years.  GA (mean ± SD): 34.9 ± 1.9.  BW (mean ± SD): 2350 ± 470.5 g. | CONTROLS:  Sample (N, sex): 19, (47% male).  Age (mean ± SD): 8.8 ± 1.1 years.  GA (mean ± SD): 36.8 ± NR.  BW (mean ± SD): NR. | CRF:  Maximal incremental cardiopulmonary exercise test (CPET). | No differences in the relevant outcomes. | |
| Welsh et al.^24^ | Case-control | CASES:  Sample (N, sex): 38, (29% male).  Age (mean ± SD): 11.1 ± 0.4 years.  GA (mean; range): 25; 24.7-25.1 weeks.  BW (mean ± SD): 740 ± 107 g. | CONTROLS:  Sample (N, sex): 38, (39% male).  Age (mean ± SD): 11.0 ± 0.5 years.  GA (mean; range): 40; 40-40.4 weeks.  BW (mean ± SD): 3360 ± 527 g. | CRF:  Cycle ergometer. | ↓ VO_2max_. | |
| Yuji Ito et al.^25^ | Case-control | CASES:  Sample (N, sex): 22, (50% male).  Age (mean): 8 years.  GA (mean; range): 36; 34-36 weeks  BW (mean; range): 2360; 1900-2840 g. | CONTROLS:  Sample (N, sex): 255, (48% male).  Age (mean): 8 years.  GA (mean; range): 39; 37-41 weeks.  BW (mean; range): 3050; 1750-4160 g. | Strength:  Dynamometer and Five Time Sit-to-stand Test. | ↓ Lower-limb strength. | |
| Abbreviations: BPD = bronchopulmonary dysplasia; BW = birth weight; CRF=Cardiorespiratory fitness; DCD = Developmental Coordination Disorder; EP= Extremely Preterm; F = female; FT = full term; GA = gestational age at birth; HBW = High Birth Weight; IBW= Insufficient Birth Weight, IQR=Interquartile Range; LBW=Low Birth Weight; M = male; MPT= Moderately Preterm; M/S= Moderate/Severe; NBW= Normal Birth Weight; NR = not reported; PT = preterm; VLBW = Very Low Birth Weight; VPT= Very Preterm; VO_2max_ = maximum oxygen consumption | | | | | |  |

**Table S4.** Summary of the studies included in the systematic review analyzing the influence of low birth weight (or birth weight as a continuous variable) on physical fitness.

| *Authors* | *Design* | *Population* | | *Outcomes (test)* | *Main results* |
| --- | --- | --- | --- | --- | --- |
| Ahlqvist et al.^26^ | Cohort. | Sample (N, sex): 286761, (100% male).  Age (mean ± SD): 18.3 ± 0.4 years.  GA (mean ± SD): 39.6 ± 1.1 weeks.  BW (mean ± SD): 3599 ± 484 g. | | CRF:  Cycle ergometer. | Greater birthweight was associated with higher VO_2max_. |
| Ahlqvist et al.^27^ | Cohort | Sample (N, sex): 144369, (100% male).  -High grip strength and LBW group: 32723, 0F/32723M.  -Low grip strength and LBW group: 39310, 0F/39310M.  -High grip strength and HBW group: 41535, 0F/41535M.  -Low grip strength and HBW group:30801, 0F/30801M  Age (mean ± SD): 18.3 ± 0.4 years.  GA (mean ± SD): 39.6 ± 1.1 weeks.  BW (mean ± SD): 3574 ± 486 g.  -High grip strength and LBW group: 3245 ± 300 g.  -Low grip strength and LBW group: 3190 ± 330 g.  -High grip strength and HBW group: 3953 ± 348 g.  -Low grip strength and HBW group: 3901 ± 329 g. | | Strength:  Dynamometer. | Strength associated with birth weight. |
| Baraldi et al.^28^ | Case-control | CASES:  Sample (N, sex): 15, (40% male).  Age (mean ± SD; range): 9.9 ± 1.8; 7.8-12.2 years.  GA (mean ± SD; range): 32.1 ± 3.0; 28-37 weeks.  BW (mean ± SD; range): 1287 ± 143; 1000-1500 g. | CONTROLS:  Sample (N, sex): 26, (NR male).  Age (mean ± SD; range): 9.7 ± 1.9; 7.3-12.8 years.  GA (mean ± SD; range): 39.9± 0.6; 37-41 weeks.  BW (mean ± SD; range): 3226 ± 167; 2140-3850 g. | CRF:  Treadmill test. | No differences in the relevant outcomes. |
| Burns et al.^29^ | Case-control | CASES:  Sample (N, sex): 54, (57% male).  Age (mean ± SD): 12.5 ± 0.7 years.  GA (mean ± SD): 26.5 ± 2.04 weeks.  BW (mean ± SD): 771 ± 148 g. | CONTROLS:  Sample (N, sex): 55, (51% male).  Age (mean ± SD): 12.4 ± 0.9 years.  GA (mean ± SD): > 37 weeks.  BW (mean ± SD): NR. | CRF:  Shuttle run test. | ↓VO_2max_. |
| Ciesla et al.^30^ | Case-control | CASES:  Sample (N, sex): 2789, (57% male).  Age (mean ± SD): 6.9 ± 0.3 years.  GA (range): 21-44 weeks.  BW (mean ± SD):  2333 ± 462 g in boys.  2406 ± 440 g in girls. | CONTROLS:  Sample (N, sex): 28008, (52% male).  Age (mean ± SD): 6.9 ± 0.3 years.  GA (range): 21-44 weeks.  BW (mean ± SD): 3514 ± 476 g in boys.  3407 ± 436 g in girls. | Strength:  Sit-up test, standing long jump test and bent arm hang test.  Flexibility:  Sit and Reach.  Agility:  10x5 m. | ↓ Flexibility, agility and strength. |
| Clemm et al.^31^ | Case-control. | CASES:  Sample (N, sex): 75, (46% male).  -1982-1985 cohort: 40, (55% male).  -1991-1992: 35, (37% male).  Age (mean ± SD): 14.2 years.  -1982-1985 cohort: 17.5 ± 1.1 years.  -1991-1992 cohort: 10.5 ± 0.4 years.  GA (mean ± SD):  1982-1985 cohort:  -Non-BPD group: 10 subjects with 28.3 ± 1.6 weeks.  - Mild BPD group: 18 subjects with 27.1 ± 1.1 weeks.  -M/S group: 12 subjects with 27.0 ± 1.2 weeks.    1991-1992 cohort:  -Non-BPD group: 9 subjects with 28.3 ± 1.4 weeks.  -Mild BPD group: 14 subjects with 26.5 ± 1.5 weeks.  -M/S group: 12 subjects with 25.8 ± 1.5 weeks.  BW (mean ± SD):  1982-1985 cohort:  -Non-BPD group: 10 subjects with 1170 ± 150 g.  -Mild BPD group: 18 subjects with 1023 ± 192 g.  -M/S group: 12 subjects with 887 ± 126 g.    1991-1992 cohort:  -Non-BPD group: 9 subjects with 1053 ± 153 g.  -Mild BPD group: 14 subjects with 927 ± 208 g.  -M/S group: 12 subjects with 851 ± 203 g. | CONTROLS:  Sample (N, sex): 75, (46% male).  -1982-1985 cohort: 40, (55% male).  -1991-1992: 35, (37% male).  Age (mean ± SD): 14.4 years.  -1982-1985 cohort: 17.7 ± 1.2 years.  -1991-1992 cohort: 10.6 ± 0.4 years.  GA (mean ± SD):  NR  BW (mean ± SD):  NR | CRF:  Treadmill test. | ↓VO_2max_ in male. |
| Clemm et al.^32^ | Case-control. | CASES:  Sample (N, sex): 34, (53% male).  -NO BPD group: 9, (44% male).  -Mild BPD group: 17, (58% male).  -M/S group: 8, (50% male).  Age (mean): 24.5 years  GA (mean ± SD; range):  -NO BPD group: 28.1± 1.2; 27-30 weeks  -Mild BPD group: 27.1 ± 1.1; 25-28 weeks.  -M/S group: 27.5 ± 1.7; 26-30 weeks.  BW (mean ± SD; range):  -NO BPD group: 1173 ± 163; 960-1480 g.  -Mild BPD group: 1019 ± 197; 580-1340 g.  -M/S group: 866 ± 127; 670-1080 g. | CONTROLS:  Sample (N, sex): 33, (52% male).  Age (mean): 25.1 years.  GA (mean): NR.  BW (mean): NR. | CRF:  Treadmill test. | No significant differences in VO_2max_. |
| Clemm et al.^33^ | Case-control | CASES:  Sample (N, sex): 26, (46% male).  -NO BPD group: 5, (40% male).  -Mild BPD group: 10, (30% male).  -M/S group: 11, (63% male).  Age (range): (10-18 years)  GA (mean ± SD; range):  -NO BPD group: 29 ± 1.4; 28-30.  -Mild BPD group: 26.7 ± 1.6; 24-28.  -M/S group: 26.1 ± 1.2; 24-28.  BW (mean ± SD; range):  -NO BPD group: 996 ± 77; 930-1126 g.  -Mild BPD group: 973 ± 215; 710-1370 g.  -M/S group: 866 ± 205; 570-1200 g. | CONTROLS:  Sample (N, sex): 22, (27% male).  Age (range): (10-18 years).  GA (mean ± SD): at term.  BW (mean ± SD; range): 3560 ± 290; 3010-3990 g. | CRF:  Treadmill test. | No significant differences in VO_2max_. |
| Crispi et al.^34^ | Case-control | CASES:  Sample (N, sex): 81, (47% male).  Age (mean): 34.4 years.  GA (mean, IQR): 40, (39-41) weeks.  BW (mean, IQR): 2570, (2440-2700) g. | CONTROLS:  Sample (N, sex): 77, (57% male).  Age (mean): 33.7 years.  GA (mean, IQR): 40, (39-40) weeks.  BW (mean, IQR): 3380, (3250-3550) g. | CRF:  Cycle ergometer. | ↓VO_2max_. |
| Evensen et al.^35^ | Case-control | CASES:  Sample (N, sex): 37, (38% male).  Age (mean): 18.2 years.  GA (mean, range): 28 (24-35).  BW (mean, range): 1245, (800-1500). | CONTROLS:  Sample (N, sex): 63, (46% male).  Age (mean): 18.6 years.  GA (mean, range): 40 (37-42).  BW (mean, range): 3700, (2670-5140). | CRF:  Treadmill test. | ↓CRF. |
| Ford et al.^36^ | Case-control | CASES:  Sample (N, sex): 24, (38% male).  Age (mean ± SD): 5.1 ± 0.2 years.  GA (mean): NR.  BW (mean; range): 1165; 750-1480 g. | CONTROLS:  Sample (N, sex): 18, (72% male).  Age (mean ± SD): 5.1 years.  GA (mean ± SD): NR.  BW (mean; range): 4366; 2800-4830 g. | Strength:  Dynamometer. | ↓ Grip strength. |
| Farrell et al.^37^ | Case-control | CASES:  Sample (N, sex): 14, (36% male)  Age (mean ±SD): 21 ± 1 years.  GA (mean ± SD): 28 ± 2 weeks.  BW (mean ± SD): 1027 ± 296 g. | CONTROLS:  Sample (N, sex): 16, (38% male)  Age (mean ± SD): 22 ± 1 years  GA (range): > 36 weeks.  BW (range): > 1500g. | CRF:  A cycle ergometer to volitional exhaustion. | ↓ VO_2max_. |
| Haraldsdottir et al.^38^ | Case-control | CASES:  Sample (N, sex): 21, (38% male)  Age (mean ±SD): 13 ± 0.7 years.  GA (mean ± SD): 27.9 ± 2.1 weeks.  BW (mean ± SD): 1097 ± 274 g. | CONTROLS:  Sample (N, sex): 20, (50% male)  Age (mean ± SD): 13.3 ± 0.7 years.  GA (mean ± SD): 39.7 ± 0.9 weeks.  BW (mean ± SD): 3497 ± 366 g. | CRF:  Cycle ergometer.  Strength:  Vertical jump and dynamometer. | ↓ VO_2max_.  No significant differences for strength. |
| Kilbride et al.^39^ | Case-control | CASES:  Sample (N, sex): 50, (32% male).  Age (mean ± SD): 11.3 ± 1.6 years.  GA (mean ± SD): 26.1 ± 1.6 weeks.  BW (mean ± SD): 701 ± 80 g. | CONTROLS:  Sample (N, sex): 25, (44% male).  Age (mean ± SD): 11.1 ± 1.3 years.  GA (mean ± SD): NR.  BW (mean): NR. | CRF:  Treadmill test. | ↓VO_2max_. |
| Kriemler et al.^9^ | Case-control | CASES:  Sample (N, sex): 14 (57% male).  Age (range): 6.1-7.8 years.  GA (mean ± SD):  28.3 ± 1.7 weeks.  BW (mean ± SD):  -1,108 ± 202 g. | CONTROLS:  Sample (N, sex): 24 (65% male).  Age (range): 5.5-7.4 years.  GA (mean ± SD): NR.  BW (mean ± SD): 1455 ± 595 g. | CRF:  Cycle ergometer. | ↓CRF. |
| Morrison et al.^40^ | Case-control | CASES:  Sample (N, sex): 95, (62% male).  Age (mean ± SD): 31.6 ± 1.6 years.  GA (mean ± SD): 27.2 ± 2.5 weeks.  BW (mean ± SD): 829 ± 131 g. | CONTROLS:  Sample (N, sex): 88, (59% male).  Age (mean ± SD): 31.9 ± 1.4 years.  GA (mean ± SD): 40.0 ± 0 weeks.  BW (mean ± SD): 3388 ± 444 g. | Strength:  Dynamometer. | ↓ Grip strength. |
| Moura dos-Santos et al.^41^ | Case-control | CASES:  Sample (N, sex): 100, (50% male).  Age (mean ± SD): 8.70 ± 1.0 years.  GA (mean ± SD): NR.  BW (mean ± SD): 2084 ± 0.3 g. | CONTROLS:  Sample (N, sex): 256, (57% male).  Age (mean ± SD): 8.9 ± 1 years.  GA (mean ± SD): NR.  BW (mean ± SD): 3426 ± 0.2 g. | Strength:  Dynamometer and curl-ups.  Flexibility:  Sit and reach.  CRF:  1-mil run/walk test.  Agility:  Square test.  Speed:  20-m run. | ↓ Strength and running speed.  No significant differences for the remaining outcomes of interest. |
| Pal et al.^42^ | Case-control | CASES:  Sample (N, sex): 189, (55% male).  Age (mean ± SD): 7 ± 1.5 years.  GA (mean ± SD): 39 ± 2.4 weeks.  BW (mean ± SD): BW 2590 ± 510 g both cohorts. | CONTROLS:  Sample (N, sex): 282, (55% male).  Age (mean ± SD): 7.2 ± 1.6 years.  GA (mean ± SD): 39.7 ± 2.1 weeks.  BW (mean ± SD): BW 2590 ± 510 g both cohorts. | Strength:  Horizontal jump and vertical jump. | ↓ Strength. |
| Rogers et al.^43^ | Case-control | CASES:  Sample (N, sex): 53, (32% male).  Age (mean; range): 17.3; 16.3-19.7 years.  GA (mean; range): 25.8; 23-29 weeks.  BW (mean; range): 719; 520-800 g. | CONTROLS:  Sample (N, sex): 31, (55% male).  Age (mean; range): 17.8; 16.5-19.0 years.  GA (mean; range): 40; 39-42 weeks.  BW (mean; range): 3506; 3068-4196 g. | CRF:  Modified Canadian Aerobic Fitness Test (mCAFT).  Strength  Dynamometer and vertical jump.  Flexibility  Sit and reach. | ↓ VO_2max_, flexibility and strength. |
| Smith et al.^44^ | Case-control | CASES:  Sample (N, sex): 126, (47% male).  Age (mean ± SD): 10.1 ± 1.1 years.  GA (mean ± SD): 26.9 ± 1.7 weeks.  BW (mean ± SD): 862 ± 161 g. | CONTROLS:  Sample (N, sex): 34, (59% male).  Age (mean ± SD): 11.6 ± 0.8 years.  GA (mean ± SD): 39.4 ± 1.2 weeks.  BW (mean ± SD): 3400 ± 513 g. | CRF:  Shuttle run test and 6-minute walk-test. | ↓ VO_2max_. |
| Tchamo et al.^45^ | Case-control | CASES:  Sample (N, sex): 76, (100% male).  -LBW group: 49, 0F/49M.  -IBW group: 27, 0F/27M.  Age (range): 19-22 years.  GA (mean ± SD): NR  BW (mean ± SD):  -LBW group: 2360 ± 270 g.  -IBW group: 2830 ± 100 g. | CONTROLS:  Sample (N, sex): 105, (100% male).  -NBW: 74, 0F/74M.  -IBW: 31, 0F/31M.  Age (range): 19-22 years.  GA (mean ± SD): at term.  BW (mean ± SD):  -NBW: 3350 ± 230 g.  -HBW: 4290 ± 320 g. | Strength:  Curl ups, standing long jump and dynamometer.  Flexibility:  Sit and reach.  Speed:  20m test.  Agility:  4x4 m test. | No differences in the relevant outcomes. |
| Victorino de Souza et al.^46^ | Cohort. | Sample (N, sex): 167, (62% male).  Age (mean ± SD): 8.98 ± 1.93 years.  GA (mean ± SD): NR.  BW (mean ± SD): 3000 ± 534 g. | | CRF:  Shuttle run test.  Strength:  Grip strength with a dynamometer and horizontal jump.  Flexibility:  Sit and Reach. | ↓VO_2max_ and strength in children with lower birth weight. |
| Yang et al.^47^ | Case-control | CASES:  Sample (N, sex): 202, (45% male).  Age (mean ± SD): 28.3 ± 1.1 years.  GA (mean ± SD): 29.3 ± 2.5 weeks.  BW (mean ± SD): 1131 ± 233 g. | CONTROLS:  Sample (N, sex): 93, (40% male).  Age (mean ± SD): 28.2 ± 0.9 years.  GA (mean): NR.  BW (mean ± SD): 3362 ± 529 g. | CRF:  Maximal incremental cardiopulmonary exercise test (CPET). | ↓VO_2max_. |

Abbreviations: BPD = bronchopulmonary dysplasia; BW = birth weight; CRF=Cardiorespiratory fitness; DCD = Developmental Coordination Disorder; EP= Extremely Preterm; F = female; FT = full term; GA = gestational age at birth; HBW = High Birth Weight; IBW= Insufficient Birth Weight, IQR=Interquartile Range; LBW=Low Birth Weight; M = male; MPT= Moderately Preterm; M/S= Moderate/Severe; NBW= Normal Birth Weight; NR = not reported; PT = preterm; VLBW = Very Low Birth Weight; VPT= Very Preterm; VO_2max_ = maximum oxygen consumption

**Table S5.** Quality of the included studies.

|  | **SELECTION** | | | | **COMPARABILITY** | **OUTCOMES** | | **TOTAL** | |  |  |
| --- | --- | --- | --- | --- | --- | --- | --- | --- | --- | --- | --- |
| **Article** | **Representativeness of the sample** | **Sample size** | **Non-respondents** | **Ascertainment of the exposure (risk factor)** | **Comparability** | **Assessment of the outcome** | **Statistical test** |  |  |  |  |
| Ahlqvist et al.^27^ | * | * | * | ** | ** | ** | * | 10 | Good |  |  |
| Ahlqvist et al.^26^ | * | * |  | * | ** | ** | * | 8 | Good |  |  |
| Baraldi et al.^28^ |  | * |  | ** | * | ** | * | 8 | Good |  |  |
| Burns et al.^48^ |  | * |  | * |  | ** | * | 5 | Poor |  |  |
| Burns et al.^29^ |  | * |  | ** |  | ** | * | 6 | Poor |  |  |
| Bruun et al.^1^ |  | * | * | ** | * | * | * | 7 | Fair |  |  |
| Callaghan et al.^49^ | * | * | * | ** | * | ** | * | 9 | Good |  |  |
| Caskey et al.^1^ | * |  | * | ** | * | ** | * | 8 | Good |  |  |
| Cheong et al.^2^ | * | * | * | ** | ** | ** | * | 10 | Good |  |  |
| Cieśla et al.^30^ | * | * | * | ** | ** | ** | * | 10 | Good |  |  |
| Clemm et al.^31^ |  | * | * | ** | * | * | * | 7 | Fair |  |  |
| Clemm et al.^32^ |  | * | * | ** | * | * | * | 7 | Fair |  |  |
| Clemm et al.^33^ |  | * | * | ** | * | * | * | 7 | Fair |  |  |
| Crispi et al.^34^ | * | * |  | ** | ** | * | * | 8 | Good |  |  |
| Cousins et al.^3^ |  | * | * | * | * | ** | * | 7 | Fair |  |  |
| Dziuba et al.^50^ |  | * |  | * |  | ** | * | 5 | Poor |  |  |
| Eshaghi et al.^51^ | * | * |  | ** | * | * | * | 7 | Fair |  |  |
| Evensen et al.^52^ |  | * | * | ** | * | ** | * | 9 | Good |  |  |
| Evensen et al.^53^ |  | * | ***** | ** |  | ** | * | 7 | Fair |  |  |
| Evensen et al.^35^ |  | * | ***** | ** | * | ** | * | 9 | Good |  |  |
| Farrell et al.^37^ | * |  | * | ** | * | ** | * | 8 | Good |  |  |
| Fitzgerald et al.^4^ | * | * |  | ** | ** | ** | * | 9 | Good |  |  |
| Ford et al.^36^ | * | * |  | ** | * | ** | * | 8 | Good |  |  |
| Forslund^54^ |  |  |  | ** | * | ** | * | 7 | Fair |  |  |
| Goss et al.^5^ | * |  |  | ** | * | ** | * | 7 | Fair |  |  |
| Guimaraes et al.^55^ | * | * | * | * | ** | ** | * | 9 | Good |  |  |
| Haraldsdottir et al.^38^ | * |  |  | ** | ** | ** | * | 9 | Good |  |  |
| Hemgren & Persson^56^ | * | * | * | ** | ** | ** | * | 10 | Good |  |  |
| Hemgren & Persson^57^ | * | * | * | ** | ** | ** | * | 10 | Good |  |  |
| Huckstep et al.^6^ | * | * |  | ** | * | * | * | 8 | Good |  |  |
| Husby et al.^58^ | * | * | * | ** | ** | ** | * | 10 | Good |  |  |
| Jenny Bolk et al.^59^ | * | * | * | ** |  | ** | * | 8 | Good |  |  |
| Jia You et al.^60^ |  |  |  | * | * | ** | * | 6 | Poor |  |  |
| Killbride et al.^39^ | * | * | * | ** | * | ** | * | 8 | Good |  |  |
| Kriemler et al.^9^ | * | * |  | * | * | ** | * | 7 | Fair |  |  |
| Kosiecz et al.^7^ | * | * |  | ** |  | * | * | 6 | Poor |  |  |
| Kristie Poole et al.^61^ | * | * |  | ** | ** | ** | * | 9 | Good |  |  |
| Lee et al.^62^ | * |  |  | ** |  | ** | * | 6 | Poor |  |  |
| Lovering et al.^8^ | * | * | * | ** | * | * | * | 8 | Good |  |  |
| Maggi et al.^63^ | * | * |  | ** | * | ** | * | 8 | Good |  |  |
| Manson et al.^64^ | * | * |  | ** | ** | ** | * | 9 | Good |  |  |
| Martínez-Zamora et al.^65^ | * | * | * | ** | ** | ** | * | 10 | Good |  |  |
| Mckay et al.^10^ | * | * |  | ** | * | ** | * | 8 | Good |  |  |
| Morrison et al.^40^ |  | * | * | ** | * | ** | * | 8 | Good |  |  |
| Moura-dos-Santos et al.^41^ | * | * | * | ** | ** | ** | * | 10 | Good |  |  |
| Narang et al.^11^ |  |  |  | ** | * | ** | * | 6 | Poor |  |  |
| Odd et al.^66^ | * | * |  | ** | ** | ** | * | 9 | Good |  |  |
| O’Dea et al.^12^ | * | * |  | ** | * | ** | * | 8 | Good |  |  |
| Oliveira et al.^67^ | * |  |  | ** |  | ** | * | 6 | Poor |  |  |
| Pal et al.^42^ | * | * | * | ** | ** | ** | * | 10 | Good |  |  |
| Pikel et al.^13^ |  |  |  | ** | * | ** | * | 6 | Poor |  |  |
| Pin et al.^68^ | * | * | * | ** | * | ** | * | 9 | Good |  |  |
| Pinheiro et al.^14^ | * |  |  | * | * | ** | * | 6 | Poor |  |  |
| Poole et al.^69^ | * | * | * | ** | * | ** | * | 9 | Good |  |  |
| Poole et al.^74^ | * |  |  | ** | ** | ** | * | 8 | Good |  |  |
| Praprotnik et al.^15^ | * | * | * | * | * | ** |  | 8 | Fair |  |  |
| Rodríguez Fernández et al.^70^ |  |  |  | ** | * | ** | * | 6 | Poor |  |  |
| Rogers et al.^43^ |  | * |  | ** | * | ** | * | 7 | Fair |  |  |
| Ruf et al.^16^ | * |  |  | ** | ** | ** | * | 8 | Good |  |  |
| Saigal et al.^71^ | * | * |  | ** | * | ** | * | 8 | Good |  |  |
| Smith et al.^44^ |  | * | * | ** | * | ** | * | 9 | Good |  |  |
| Svedenkrans et al.^17^ | * | * |  | * |  | * | * | 5 | Poor |  |  |
| Svedenkrans et al.^18^ | * | * |  | * |  | * | * | 5 | Poor |  |  |
| Svien^19^ |  | * |  | ** | * | ** | * | 7 | Fair |  |  |
| Syrengelas et al.^72^ | * | * |  | ** | * | ** | * | 8 | Good |  |  |
| Tchamo et al.^45^ |  | * | * | ** | * | ** | * | 8 | Good |  |  |
| Tikanmaki et al.^20^ | * | * |  | * | ** | ** | * | 8 | Good |  |  |
| Toome et al.^73^ | * | * |  | ** | ** | ** | * | 9 | Good |  |  |
| Van Deutekom et al.^21^ | * | * |  | ** | ** | ** | * | 9 | Good |  |  |
| Victorino de Souza et al.^46^ | * |  |  | ** | ** | ** | * | 8 | Good |  |  |
| Vrijlandt et al.^22^ | * | * | * | ** | ** | ** | * | 10 | Good |  |  |
| Van Hus et al.^75^ | * | * | * | ** | ** | ** | * | 10 | Good |  |  |
| Waetge et al.^76^ | * | * |  | ** | * | * | * | 7 | Fair |  |  |
| Weigelt et al. ^23^ | * | * |  | ** | * | * | * | 7 | Fair |  |  |
| Welsh et al.^24^ | | |  |  |  | ** | ** | ** | * | 7 | Fair |
| Yang et al.^47^ | | * | * | * | ** | ** | * | * | 9 | Good |  |
| Yuji Ito et al.^25^ | | * | * | * | ** | * | * | * | 8 | Good |  |

**Fig. S1**. Forest plot displaying the effect of preterm birth on cardiorespiratory fitness.

**Fig. S2.** Forest plot displaying the effect of low birth weight on cardiorespiratory fitness.

**Fig. S3.** Forest plot displaying the effect of preterm birth on muscle strength.

**
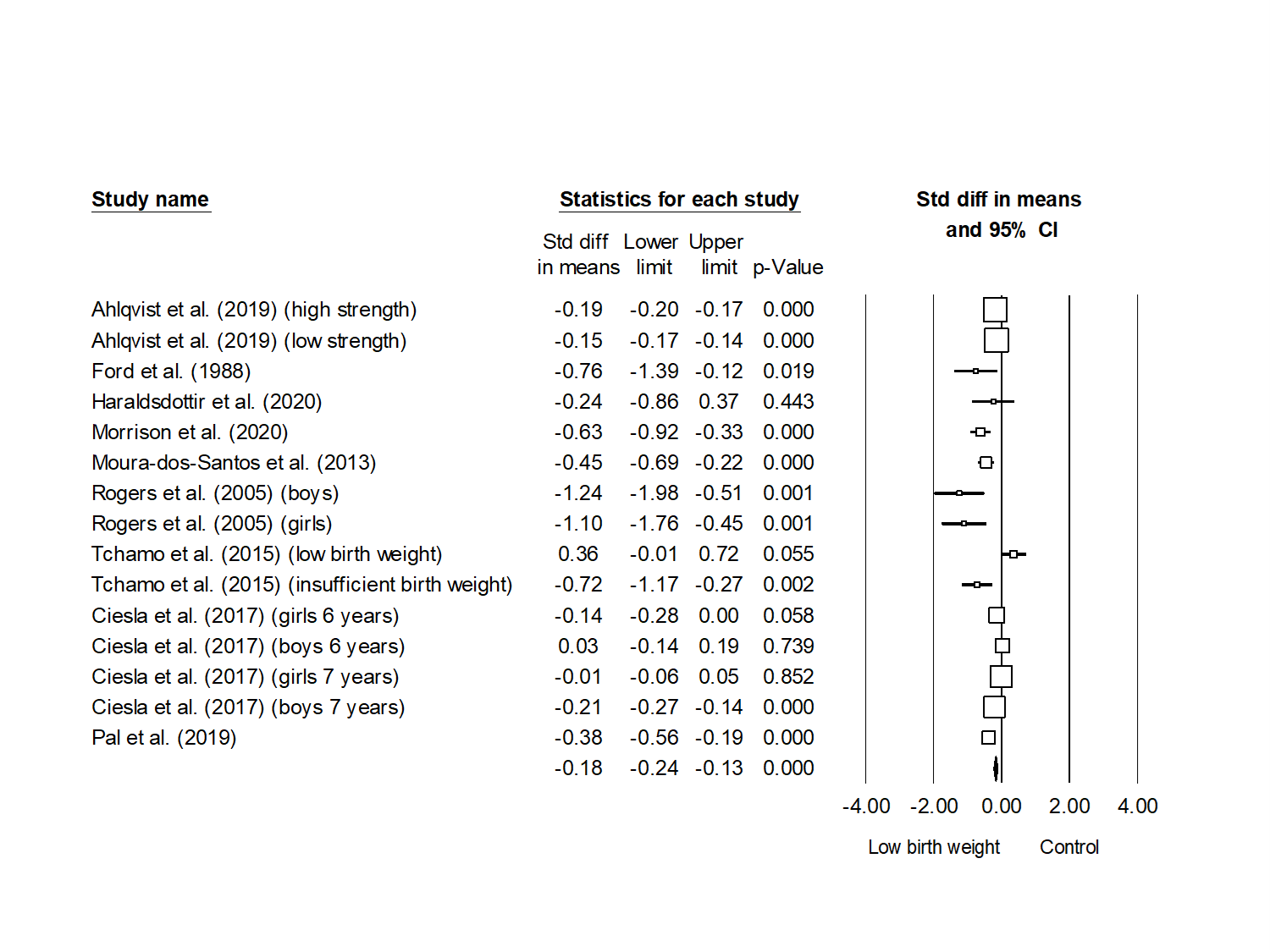
**

**Fig. S4.** Forest plot displaying the effect of low birth weight on muscle strength.

**
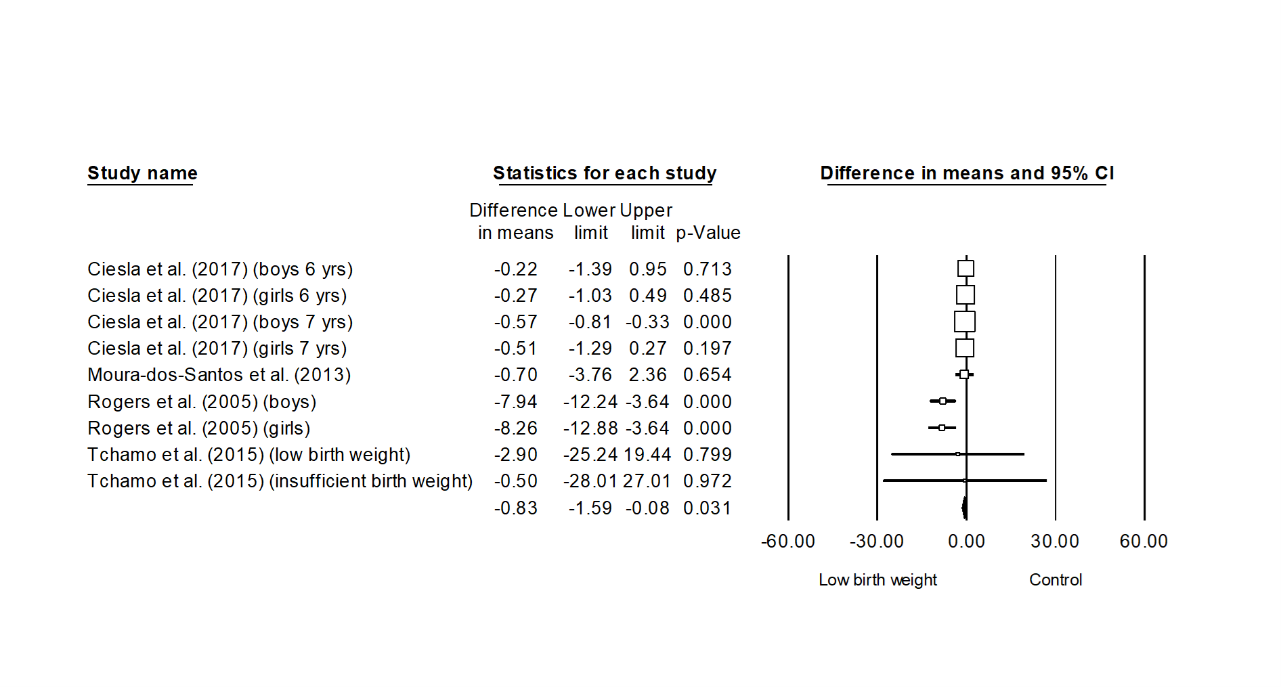
**

**Fig. S5.** Forest plot displaying the effect of low birth weight on flexibility.

**
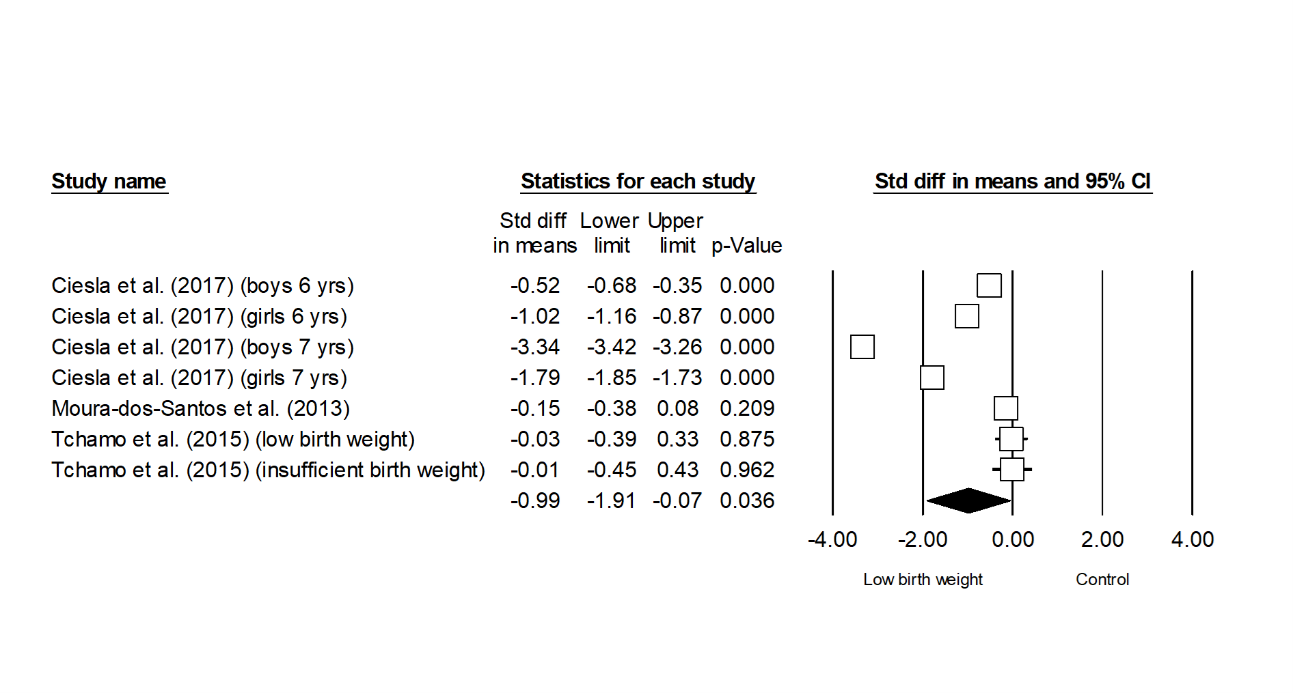
**

**Fig. S6.** Forest plot displaying the effect of low birth weight on agility.

**Fig. S7.** Meta-regression analysis displaying the association between gestational age and the magnitude of impairment in cardiorespiratory fitness in children born preterm. A significant association was found (p=0.028).

**Fig. S8.** Meta-regression analysis displaying the association between birth weight and the magnitude of impairment in cardiorespiratory fitness in children born with low birth weight. A trend towards a significant association was found (p=0.058).

**Fig. S9.** Meta-regression analysis displaying the association between gestational age and the magnitude of impairment in muscle strength in children born preterm. A significant association was found (p=0.024).

**
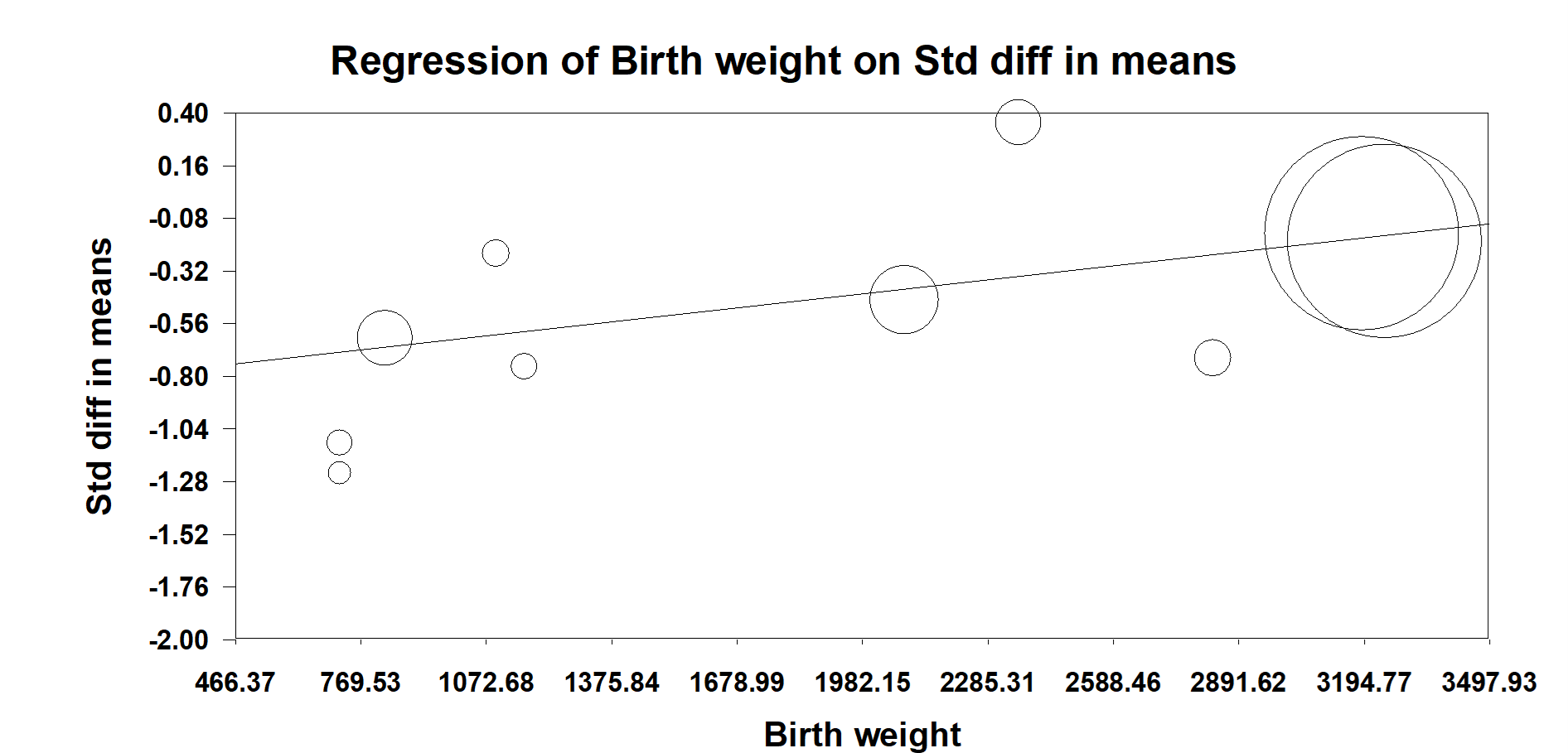
**

**Fig. S10.** Meta-regression analysis displaying the association between birth weight and the magnitude of impairment in muscle strength in children born with low birth weight. A significant association was found (p<0.001).

**Fig. S11.** Meta-regression analysis displaying the association between age at assessment and the magnitude of impairment in cardiorespiratory fitness in individuals born preterm. No significant association was found (p=0.069).

**Fig. S12.** Meta-regression analysis displaying the association between age at assessment and the magnitude of impairment in cardiorespiratory fitness in individuals born with low birth weight. No significant association was found (p=0.490).

**Fig. S13.** Meta-regression analysis displaying the association between age at assessment and the magnitude of impairment in muscle strength in individuals born preterm. No significant association was found (p=0.892).

**Fig. S14.** Meta-regression analysis displaying the association between age at assessment and the magnitude of impairment in muscle strength in individuals born with low birth weight. No significant association was found (p=0.960).

**References cited in Supplementary Files**

1. Caskey S, Gough A, Rowan S, et al. Structural and functional lung impairment in adult survivors of bronchopulmonary dysplasia. *Ann Am Thorac Soc*. 2016;13(8):1262-1270. doi:10.1513/AnnalsATS.201509-578OC

2. Cheong JLY, Haikerwal A, Wark JD, et al. Cardiovascular Health Profile at Age 25 Years in Adults Born Extremely Preterm or Extremely Low Birthweight. *Hypertension*. Published online 2020:1838-1846. doi:10.1161/HYPERTENSIONAHA.120.15786

3. Cousins M, Hart K, Williams EM, Kotecha S. Impaired exercise outcomes with significant bronchodilator responsiveness in children with prematurity-associated obstructive lung disease. *Pediatr Pulmonol*. 2022;57(9):2161-2171. doi:10.1002/ppul.26019

4. FitzGerald TL, Cameron KL, Albesher RA, et al. Strength, Motor Skills, and Physical Activity in Preschool-Aged Children Born Either at Less Than 30 Weeks of Gestation or at Term. *Phys Ther*. 2021;101(5):1-10. doi:10.1093/ptj/pzab037

5. Goss KN, Beshish AG, Barton GP, et al. Early pulmonary vascular disease in young adults born preterm. *Am J Respir Crit Care Med*. 2018;198(12):1549-1558. doi:10.1164/rccm.201710-2016OC

6. Huckstep OJ, Burchert H, Williamson W, et al. Impaired myocardial reserve underlies reduced exercise capacity and heart rate recovery in preterm-born young adults. *Eur Heart J Cardiovasc Imaging*. 2021;22(5):572-580. doi:10.1093/ehjci/jeaa060

7. Kosiecz A, Chrościńska-Krawczyk M, Taczała J, Zawadka M. Evaluation of physical and cardiorespiratory fitness in 7-year-old prematurely born children – preliminary study. *Ann Agric Environ Med*. 2021;28(3):502-508. doi:10.26444/aaem/127220

8. Lovering AT, Laurie SS, Elliott JE, et al. Normal pulmonary gas exchange efficiency and absence of exercise-induced arterial hypoxemia in adults with bronchopulmonary dysplasia. *J Appl Physiol*. 2013;115(7):1050-1056. doi:10.1152/japplphysiol.00592.2013

9. Martinez-Zamora MD, Valenzuela PL, Esteban Díez I, Martínez-de-Quel Ó. Influence of preterm birth on physical fitness in early childhood. *Eur J Sport Sci*. 2023;(May):1-10. doi:10.1080/17461391.2023.2207082

10. McKay L, Goss KN, Haraldsdottir K, et al. Decreased ventricular size and mass mediate the reduced exercise capacity in adolescents and adults born premature. *Early Hum Dev*. 2021;160(July):105426. doi:10.1016/j.earlhumdev.2021.105426

11. Narang BJ, Manferdelli G, Kepic K, et al. Effects of Pre-Term Birth on the Cardio-Respiratory Responses to Hypoxic Exercise in Children. Published online 2022.

12. O’Dea CA, Logie K, Wilson AC, et al. Lung abnormalities do not influence aerobic capacity in school children born preterm. *Eur J Appl Physiol*. 2021;121(2):489-498. doi:10.1007/s00421-020-04530-2

13. Robič Pikel T, Starc G, Strel J, Kovač M, Babnik J, Golja P. Impact of prematurity on exercise capacity and agility of children and youth aged 8 to 18. *Early Hum Dev*. 2017;110(April):39-45. doi:10.1016/j.earlhumdev.2017.04.015

14. Tsopanoglou S, Davidson J, Dourado V, Goulart A, Barros M, dos Santos A. Aerobic Capacity of Preterm Children with Very Low Birth Weight at School Age and its Associated Factors. *Arch Bronconeumol (English Ed*. 2020;56(3):157-162. doi:10.1016/j.arbr.2019.05.022

15. Praprotnik M, Stucin Gantar I, Krivec U, Lucovnik M, Rodman Berlot J, Starc G. Physical fitness trajectories from childhood to adolescence in extremely preterm children: A longitudinal cohort study. *Pediatr Pulmonol*. 2023;58(7):1904-1911. doi:10.1002/ppul.26410

16. Ruf K, Thomas W, Brunner M, Speer CP, Hebestreit H. Diverging effects of premature birth and bronchopulmonary dysplasia on exercise capacity and physical activity - A case control study. *Respir Res*. 2019;20(1):1-9. doi:10.1186/s12931-019-1238-0

17. Svedenkrans J, Kowalski J, Norman M, Bohlin K. Low exercise capacity increases the risk of low cognitive function in healthy young men born preterm: A population-based cohort study. *PLoS One*. 2016;11(8):1-12. doi:10.1371/journal.pone.0161314

18. Svedenkrans J, Henckel E, Kowalski J, Norman M, Bohlin K. Long-term impact of preterm birth on exercise capacity in healthy young men: A national population-based cohort study. *PLoS One*. 2013;8(12). doi:10.1371/journal.pone.0080869

19. Svien L. Health-Related Fitness of Seven-to-10-Year-Old Children with Histories of Preterm Birth. Published online 2003:74-83.

20. Tikanmaki M, Tammelin T, Sipola-Leppänen M, et al. Physical fitness in young adults born preterm. *Pediatrics*. 2016;137(1). doi:10.1542/peds.2015-1289

21. Van Deutekom AW, Chinapaw MJM, Vrijkotte TGM, Gemke RJBJ. The association of birth weight and infant growth with physical fitness at 8-9 years of age - The ABCD study. *Int J Obes*. 2015;39(4):593-600. doi:10.1038/ijo.2014.204

22. Vrijlandt E, Gerritsen J, Boezen H, Grevink R, Duiverman E. Lung function and exercise capacity in young adults born prematurely. *Am J Respir Crit Care Med*. 2006;173(8):890-896. doi:10.1164/rccm.200507-1140OC

23. Weigelt A, Bleck S, Huebner MJ, et al. Impact of premature birth on cardiopulmonary function in later life. *Eur J Pediatr*. 2023;182(7):3265-3274. doi:10.1007/s00431-023-04952-y

24. Welsh L, Kirkby J, Lum S, et al. The EPICure study: Maximal exercise and physical activity in school children born extremely preterm. *Thorax*. 2010;65(2):165-171. doi:10.1136/thx.2008.107474

25. Ito Y, Ito T, Sugiura H, et al. Physical functions and gait performance in school-aged children born late preterm. *Early Hum Dev*. 2021;163(July):105478. doi:10.1016/j.earlhumdev.2021.105478

26. Ahlqvist VH, Persson M, Ortega FB, Tynelius P, Magnusson C, Berglind D. Birth Weight and Cardiorespiratory Fitness Among Young Men Born at Term: The Role of Genetic and Environmental Factors. *J Am Heart Assoc*. 2020;9(3). doi:10.1161/JAHA.119.014290

27. Ahlqvist V, Persson M, Ortega FB, Tynelius P, Magnusson C, Berglind D. Birth weight and grip strength in young Swedish males: a longitudinal matched sibling analysis and across all body mass index ranges. *Sci Rep*. 2019;9(1):1-8. doi:10.1038/s41598-019-46200-0

28. Baraldi E, Zanconato S, Zorzi C, Santuz P, Benini F, Zaccheilo F. Exercise performance in very low birth weight children at the age of 7-12 years. *Eur J Pediatr*. 1991;1991:713-716.

29. Burns Y, Danks M, O’Callaghan M, et al. Motor coordination difficulties and physical fitness of extremely-low-birthweight children. *Dev Med Child Neurol*. 2009;51(2):136-142. doi:10.1111/j.1469-8749.2008.03118.x

30. Cieśla E, Zaręba M, Kozieł S. The level of physical fitness in children aged 6–7 years with low birthweight. *Early Hum Dev*. 2017;111(May):23-29. doi:10.1016/j.earlhumdev.2017.05.008

31. Clemm H, Røksund O, Thorsen E, Eide G, Markestad T, Halvorsen T. Aerobic capacity and exercise performance in young people born extremely preterm. *Pediatrics*. 2012;129(1). doi:10.1542/peds.2011-0326

32. Clemm H, Vollsæter M, Røksund O, Eide G, Markestad T, Halvorsen T. Exercise capacity after extremely preterm birth: Development from adolescence to adulthood. *Ann Am Thorac Soc*. 2014;11(4):537-545. doi:10.1513/AnnalsATS.201309-311OC

33. Clemm H, Vollsæter M, Røksund O, Markestad T, Halvorsen T. Adolescents who were born extremely preterm demonstrate modest decreases in exercise capacity. *Acta Paediatr Int J Paediatr*. 2015;104(11):1174-1181. doi:10.1111/apa.13080

34. Crispi F, Rodríguez-López M, Bernardino G, et al. Exercise Capacity in Young Adults Born Small for Gestational Age. *JAMA Cardiol*. 2021;6(11):1308-1316. doi:10.1001/jamacardio.2021.2537

35. Evensen KAI, Steinshamn S, Tjønna AE, et al. Effects of preterm birth and fetal growth retardation on cardiovascular risk factors in young adulthood. *Early Hum Dev*. 2009;85(4):239-245. doi:10.1016/j.earlhumdev.2008.10.008

36. Ford GW, Kitchen WH, Doyle LW. Muscular strength at 5 years of children with a birthweight under 1500g. *J Paediatr Child Health*. 1988;24(5):295-296. doi:10.1111/j.1440-1754.1988.tb01366.x

37. Farrell E, Bates M, Pegelow D, et al. Pulmonary gas exchange and exercise capacity in adults born preterm. *Ann Am Thorac Soc*. 2015;12(8):1130-1137. doi:10.1513/AnnalsATS.201410-470OC

38. Haraldsdottir K, Watson A, Pegelow D, et al. Blunted cardiac output response to exercise in adolescents born preterm. *Eur J Appl Physiol*. 2020;120(11):2547-2554. doi:10.1007/s00421-020-04480-9

39. Kilbride H, Gelatt M, Sabath R. Pulmonary function and exercise capacity for elbw survivors in preadolescence: Effect of neonatal chronic lung disease. *J Pediatr*. 2003;143(4):488-493. doi:10.1067/S0022-3476(03)00413-X

40. Morrison K, Gunn E, Guay S, Obeid J, Schmidt L, Saigal S. Grip strength is lower in adults born with extremely low birth weight compared to term-born controls. *Pediatr Res*. 2021;89(4):996-1003. doi:10.1038/s41390-020-1012-5

41. Moura-Dos-Santos M, Wellington-Barros J, Brito-Almeida M, Manhães-de-Castro R, Maia J, Góis Leandro C. Permanent deficits in handgrip strength and running speed performance in low birth weight children. *Am J Hum Biol*. 2013;25(1):58-62. doi:10.1002/ajhb.22341

42. Pal A, Manna S, Dhara PC. Comparison between the motor function of school-aged children with normal birth weight and children with low birth weight: A cross-sectional study. *Turk J Pediatr*. 2019;61(3):374-385. doi:10.24953/turkjped.2019.03.009

43. Rogers M, Fay T, Whitfield M, Tomlinson J, Grunau R. Aerobic capacity, strength, flexibility, and activity level in unimpaired extremely low birth weight (≤800 g) survivors at 17 years of age compared with term-born control subjects. *Pediatrics*. 2005;116(1). doi:10.1542/peds.2004-1603

44. Smith L, Van Asperen P, McKay K, Selvadurai H, Fitzgerald D. Reduced exercise capacity in children born very preterm. *Pediatrics*. 2008;122(2). doi:10.1542/peds.2007-3657

45. Tchamo M, Santos M, Almeida M, Silva A, Leandro C. Physical Fitness and Birth Weight in Young Men From Maputo City, Mozambique. *Rev Bras Med do Esporte*. 2016;22(1):66-70. doi:10.1590/1517-869220162201147126

46. de Souza LV, de Meneck F, Parizotto GP, Franco M. Low birth weight and its relation to physical fitness parameters in children: Its negative effect on muscle strength and cardiorespiratory endurance. *Am J Hum Biol*. 2022;34(1):1-8. doi:10.1002/ajhb.23595

47. Yang J, Epton MJ, Harris SL, et al. Reduced Exercise Capacity in Adults Born at Very Low Birth Weight A Population-based Cohort Study. *Am J Respir Crit Care Med*. 2022;205(1):88-98. doi:10.1164/rccm.202103-0755OC

48. Burns YR, Ensbey R, O’Callaghan M. Motor abilities at eight to ten years of children born weighing less than 1000 g. *Physiotherapy*. 1999;85(7):360-369. doi:10.1016/S0031-9406(05)67189-1

49. O’Callaghan MJ, Burns Y, Gray P, et al. Extremely low birth weight and control infants at 2 years corrected age: A comparison of intellectual abilities, motor performance, growth and health. *Early Hum Dev*. 1995;40(2):115-125. doi:10.1016/0378-3782(94)01597-I

50. Dziuba E, Drzał-Grabiec J, Truszczyńska-Baszak A, Guzek K, Zajkiewicz K. Balance in children born prematurely currently aged 6-7. *Biomed Hum Kinet*. 2017;9(1):181-186. doi:10.1515/bhk-2017-0025

51. Eshaghi Z, Jafari Z, Jalaie S. Static balance function in children with a history of preterm birth. *Med J Islam Repub Iran*. 2015;29(1):1278-1286.

52. Evensen KAI, Vik T, Helbostad J, Indredavik MS, Kulseng S, Brubakk AM. Motor skills in adolescents with low birth weight. *Arch Dis Child Fetal Neonatal Ed*. 2004;89(5):451-455. doi:10.1136/adc.2003.037788

53. Evensen KAI, Skranes J, Brubakk AM, Vik T. Predictive value of early motor evaluation in preterm very low birth weight and term small for gestational age children. *Early Hum Dev*. 2009;85(8):511-518. doi:10.1016/j.earlhumdev.2009.04.007

54. Forslund M. Growth and motor performance in preterm children at 8 years of age. *Acta Paediatr Int J Paediatr*. 1992;81(10):840-842. doi:10.1111/j.1651-2227.1992.tb12115.x

55. Guimarães CLN, Reinaux CM, Botelho ACG, Lima GMS, Cabral Filho JE. Motor development evaluated by Test of Infant Motor Performance: comparison between preterm and full-term infants. *Brazilian J Phys Ther*. 2011;15(5):357-363. doi:10.1590/s1413-35552011005000021

56. Hemgren E, Persson K. Motor performance and behaviour in preterm and full-term 3-year-old children. *Child Care Health Dev*. 2002;28(3):219-226. doi:10.1046/j.1365-2214.2002.00265.x

57. Hemgren E, Persson K. Quality of motor performance in preterm and full-term 3-year-old children. *Child Care Health Dev*. 2004;30(5):515-527. doi:10.1111/j.1365-2214.2004.00439.x

58. Husby IM, Skranes J, Olsen A, Brubakk AM, Evensen KAI. Motor skills at 23years of age in young adults born preterm with very low birth weight. *Early Hum Dev*. 2013;89(9):747-754. doi:10.1016/j.earlhumdev.2013.05.009

59. Bolk J, Farooqi A, Hafström M, Aden U, Serenius F. Developmental coordination disorder and its association with developmental comorbidities at 6.5 years in apparently healthy children born extremely preterm. *JAMA Pediatr*. 2018;172(8):765-774. doi:10.1001/jamapediatrics.2018.1394

60. You J, Yang HJ, Hao MC, Zheng JJ. Late preterm infants’ social competence, motor development, and cognition. *Front Psychiatry*. 2019;10(FEB):1-8. doi:10.3389/fpsyt.2019.00069

61. Poole KL, Schmidt LA, Missiuna C, Saigal S, Boyle MH, Van Lieshout RJ. Motor coordination and mental health in extremely low birth weight survivors during the first four decades of life. *Res Dev Disabil*. 2015;43-44:87-96. doi:10.1016/j.ridd.2015.06.004

62. Lee SYR, Chow CB, Ma PYA, Ho YB, Shek CC. Gross motor skills of premature, Very low-birthweight Chinese children. *Ann Trop Paediatr*. 2004;24(2):179-183. doi:10.1179/027249304225013466

63. Maggi EF, Magalhães LC, Campos AF, Bouzada MCF. Preterm children have unfavorable motor, cognitive, and functional performance when compared to term children of preschool age. *J Pediatr (Rio J)*. 2014;90(4):377-383. doi:10.1016/j.jped.2013.10.005

64. Mercier K, Whitley MA, Manson M. Middle School Assessments that Contribute to Literacy Goals. *Strateg A J Phys Sport Educ*. 2014;27(3):22-27. doi:http://dx.doi.org/10.1080/08924562.2014.900464

65. Martinez-Zamora MD, Valenzuela PL, Díez IE, Martínez-de-Quel Ó. Influence of preterm birth on physical fitness in early childhood. *Eur J Sport Sci*. Published online April 24, 2023:1-27. doi:10.1080/17461391.2023.2207082

66. Odd DE, Lingam R, Emond A, Whitelaw A. Movement outcomes of infants born moderate and late preterm. *Acta Paediatr Int J Paediatr*. 2013;102(9):876-882. doi:10.1111/apa.12320

67. Oliveira GE, Magalhães LC, Salmela LFT. Relationship between very low birth weight, environmental factors, and motor and cognitive development of children of 5 and 6 years old. *Rev Bras Fisioter*. 2011;15(2):138-145. doi:10.1590/s1413-35552011000200009

68. Pin TW, Darrer T, Eldridge B, Galea MP. Motor development from 4 to 8 months corrected age in infants born at or less than 29 weeks’ gestation. *Dev Med Child Neurol*. 2009;51(9):739-745. doi:10.1111/j.1469-8749.2009.03265.x

69. Poole KL, Schmidt LA, Missiuna C, Saigal S, Boyle MH, Van Lieshout RJ. Motor Coordination Difficulties in Extremely Low Birth Weight Survivors Across Four Decades. *J Dev Behav Pediatr*. 2015;36(7):521-528. doi:10.1097/DBP.0000000000000199

70. Rodríguez Fernández C, Mata Zubillaga D, Rodríguez Fernández L, et al. Valoración de la coordinación y el equilibrio en niños prematuros. *An Pediatr*. 2016;85(2):86-94. doi:10.1016/j.anpedi.2015.10.009

71. Saigal S, Szatmari P, Rosenbaum P, Campbell D, King S. Cognitive abilities and school performance of extremely low birth weight children and matched term control children at age 8 years: A regional study. *J Pediatr*. 1991;118(5):751-760. doi:10.1016/S0022-3476(05)80043-5

72. Syrengelas D, Kalampoki V, Kleisiouni P, et al. Alberta Infant Motor Scale (AIMS) performance of greek preterm infants: Comparisons with full-term infants of the same nationality and impact of prematurity-related morbidity factors. *Phys Ther*. 2016;96(7):1102-1108. doi:10.2522/ptj.20140494

73. Toome L, Varendi H, Männamaa M, Vals MA, Tänavsuu T, Kolk A. Follow-up study of 2-year-olds born at very low gestational age in Estonia. *Acta Paediatr Int J Paediatr*. 2013;102(3):300-307. doi:10.1111/apa.12091

74. Poole KL, Islam UA, Schmidt LA, et al. Childhood Motor Function, Health Related Quality of Life and Social Functioning among Emerging Adults Born at Term or Extremely Low Birth Weight. *J Dev Phys Disabil*. 2017;29(3):369-383. doi:10.1007/s10882-016-9530-0

75. Van Hus JW, Potharst ES, Jeukens-Visser M, Kok JH, Van Wassenaer-Leemhuis AG. Motor impairment in very preterm-born children: Links with other developmental deficits at 5 years of age. *Dev Med Child Neurol*. 2014;56(6):587-594. doi:10.1111/dmcn.12295

76. de Godoy VCWP, de Souza FIS, Johnston C, Strufaldi MWL. Motor development of infants (6–12 months) with low birth weight. *Rev Assoc Med Bras*. 2021;67(4):529-535. doi:10.1590/1806-9282.20200966
